# Foundry-fabricated dual-color nanophotonic neural probes for photostimulation and electrophysiological recording

**DOI:** 10.1117/1.NPh.12.2.025002

**Published:** 2025-03-28

**Authors:** David A. Roszko, Fu-Der Chen, John Straguzzi, Hannes Wahn, Alec Xu, Blaine McLaughlin, Xinxin Yin, Hongyao Chua, Xianshu Luo, Guo-Qiang Lo, Joshua H. Siegle, Joyce K. S. Poon, Wesley D. Sacher

**Affiliations:** a Max Planck Institute of Microstructure Physics, Halle (Saale), Germany; b University of Toronto, Department of Electrical and Computer Engineering, Toronto, Ontario, Canada; c Max Planck-University of Toronto Centre for Neural Science and Technology, Toronto, Ontario, Canada; d Allen Institute for Neural Dynamics, Seattle, Washington, United States; e Advanced Micro Foundry Pte Ltd., Singapore

**Keywords:** neurophotonics, neural probe, nanophotonics, integrated photonics, optogenetics

## Abstract

**Significance:**

Compact tools capable of delivering multicolor optogenetic stimulation to deep tissue targets with sufficient span, spatiotemporal resolution, and optical power remain challenging to realize. Here, we demonstrate foundry-fabricated nanophotonic neural probes for blue and red photostimulation and electrophysiological recording, which use a combination of spatial multiplexing and on-shank wavelength demultiplexing to increase the number of on-shank emitters.

**Aim:**

We demonstrate silicon (Si) photonic neural probes with 26 photonic channels and 26 recording sites, which were fabricated on 200-mm diameter wafers at a commercial Si photonics foundry. Each photonic channel consists of an on-shank demultiplexer and separate grating coupler emitters for blue and red light, for a total of 52 emitters.

**Approach:**

We evaluate neural probe functionality through bench measurements and *in vivo* experiments by photostimulating through 16 of the available 26 emitter pairs.

**Results:**

We report neural probe electrode impedances, optical transmission, and beam profiles. We validated a packaged neural probe in optogenetic experiments with mice sensitive to blue or red photostimulation.

**Conclusions:**

Our foundry-fabricated nanophotonic neural probe demonstrates dense dual-color emitter integration on a single shank for targeted photostimulation. Given its two emission wavelengths, high emitter density, and long site span, this probe will facilitate experiments involving bidirectional circuit manipulations across both shallow and deep structures simultaneously.

## 1 Introduction

Optogenetics has enabled neuroscientists to selectively excite, inhibit, and modulate neural activity in a cell type–specific, wavelength-selective manner. Today, a wide range of optogenetic actuators are available, with peak activation wavelengths across the visible spectrum.^1^^–^^8^ In combination, these optogenetic actuators can enable diverse experimental designs, as opsins with distinct activation spectra (i.e., blue and red light–activated opsins) can be used for independent excitation and inhibition of different cell types within the same region^9^ or excitation and inhibition of the same cell type.^8^

To facilitate optogenetic experiments, it is crucial to develop tools that can deliver multicolor photostimulation with high spatiotemporal resolution while simultaneously recording evoked neural activity. For delivering light into superficial brain regions (depth: ≤1  mm), methods based on two-photon microscopy have been widely used.^10^^,^^11^ However, to interrogate deeper neural tissue (depth: >1  mm), researchers have developed implantable tools based on integrated optoelectronic and photonic technologies. These approaches are based on microchips consisting of (1) long (3 to 10 mm), narrow, implantable shanks with integrated optoelectronic or photonic emitters for photostimulation and (2) a larger base region with circuitry and electrical/optical interfaces. Each of these technologies exhibits notable trade-offs in terms of average emitter power, emitter numbers, and on-shank emitter densities.

Optoelectronic neural probes with high-density on-shank integrated micro light-emitting diodes (μLEDs) or organic light-emitting diodes (OLEDs) have been developed for dual-color photostimulation and neural recording.^12^^–^^14^ Recent reports have demonstrated high emitter counts (16 red and 16 blue μLEDs per shank in Ref. 12 and 256 orange or blue OLEDs per shank in Ref. 13). However, these designs either have large shank dimensions (200  μm wide in Ref. 12), which may cause excess tissue damage, or low output powers (40 to 100 nW in Ref. 13), which necessitates using multiple emitters for photostimulation with most opsins—effectively reducing the spatial resolution of the device. In addition, designs typically leverage custom fabrication steps beyond those used for complementary metal-oxide-semiconductor (CMOS) electronics, posing challenges for scaling manufacturing volumes.

Photonic neural probes guide light from external laser sources into the brain via integrated optical waveguides and diffractive (grating coupler) emitters. By delocalizing the light source from the shank and surrounding brain tissue, photonic probes offer the opportunity for high output powers beyond those of optoelectronic probes, which are limited by on-shank emitter heating. This approach also leverages the increasing maturity of silicon (Si) integrated photonics technology, charting a path toward high emitter counts on narrow shanks using dense arrays of nanophotonic waveguides in addition to scalable manufacturing volumes via commercial Si photonics foundries. Recent reports of photonic probes most often rely on silicon nitride (SiN) waveguides,^15^^–^^20^ which are commonly used in standard Si photonics^21^ and can be engineered for broadband transparency extending to the visible spectrum.^19^^,^^22^ This broadband transparency is of particular utility for multiplexing multiple photostimulation wavelengths onto each waveguide.

Currently, photonic neural probe technology is in its early development stages, with low channel counts relative to their optoelectronic probe counterparts and few reports of dual-color photostimulation functionalities. An early example of a dual-color photonic probe used silicon oxynitride waveguides to guide 405- and 635-nm light, but the large waveguide cross-sectional areas ($7\times 30\;\mu m^{2}$) limited the design to one emitter per shank.^23^ More recent demonstrations with sub-micron SiN waveguides have achieved higher channel counts. In Ref. 17, three red and three blue emitters were integrated onto a single shank and coupled to a single bus waveguide via ring resonator wavelength demultiplexer devices. Tuning of the input wavelength enabled emitter selection (wavelength-division multiplexing). In this approach, scaling of the number of emitters (and ring resonators) is expected to be limited by the inherent fabrication variation sensitivity of ring resonator devices.^24^ In Refs. 19 and 20, 14 red and 14 blue emitters were integrated onto the shank of a CMOS-based neural probe, with each emitter coupled to a separate waveguide. Emitters were selected by an on-chip photonic switching circuit integrated onto the base of the probe (spatial multiplexing). With spatial multiplexing alone, emitter densities are limited by parasitic optical coupling/crosstalk among adjacent waveguides.

**Fig. 1:**
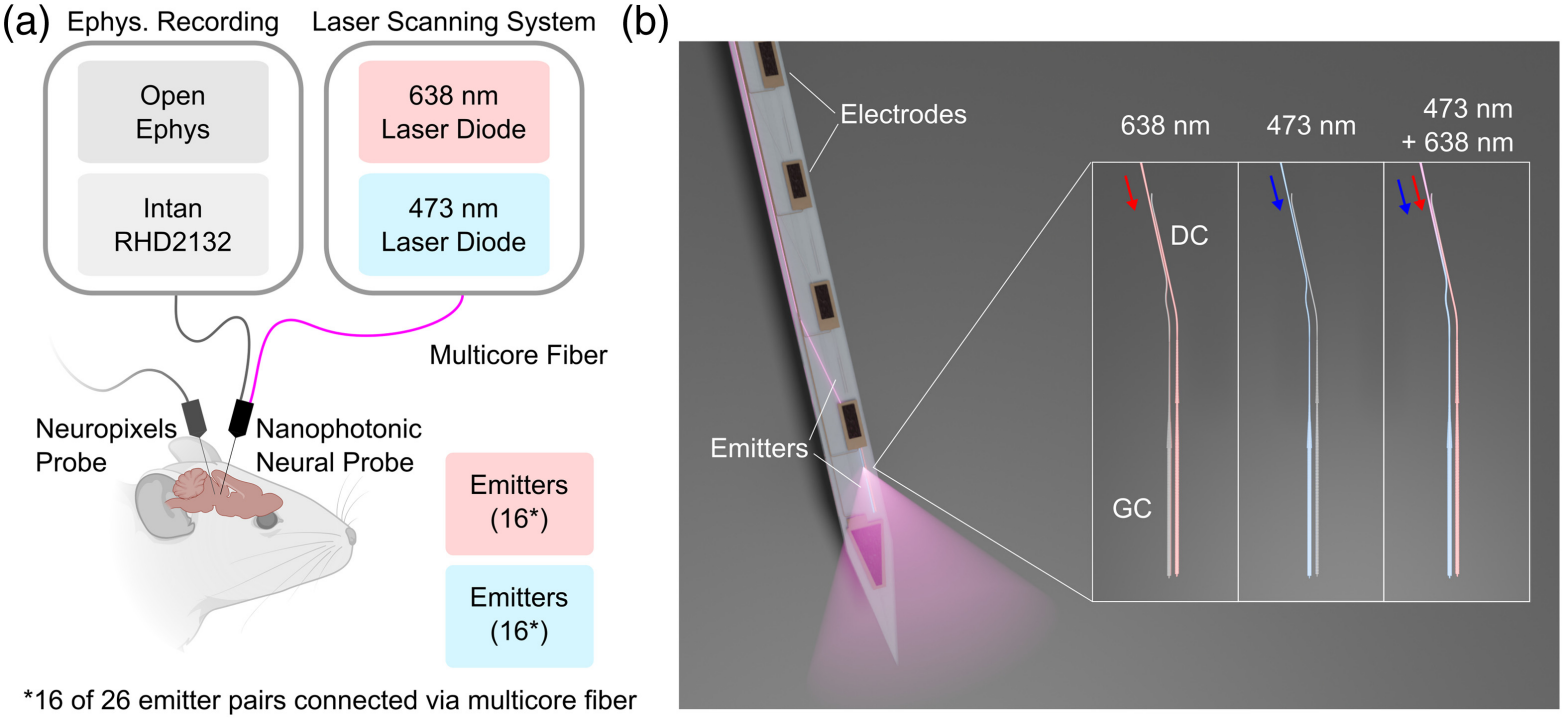
(a) Conceptual figure illustrating simultaneous recordings with a dual-color nanophotonic neural probe and a Neuropixels probe.^25^ Figure created with Ref. 26. (b) Graphical rendering of a dual-color nanophotonic neural probe. Each routing waveguide connects to a directional coupler which demultiplexes red (638 nm) and blue (473 nm) light to separate grating coupler emitters.

Here, we present dual-color photonic neural probes with combined spatial multiplexing and wavelength-division multiplexing for increasing the number of addressable emitters on a single shank. Our design, which builds on previous single-wavelength integrated probe designs from our group,^18^ is manufactured using wafer-scale processes at a commercial Si photonics foundry (Advanced Micro Foundry, Singapore), paving the way for mass production and broad dissemination within the neuroscience community. Each neural probe has 52 emitters on a single shank (26 blue and 26 red), with emitter addressing via both spatial multiplexing (with external laser scanning optics addressing a multicore fiber (MCF) coupled to the probe) and wavelength multiplexing (with red and blue input lasers). An array of spatially addressed, fiber-to-chip edge couplers and waveguides—each coupled to a core of the multicore fiber—route multiplexed blue (473 nm) and red (638 nm) light to on-shank evanescent directional-coupler wavelength demultiplexers, which direct each color to separate (red- or blue-designed) grating coupler emitters. In addition, our design contains 26 recording electrodes for electrophysiological recording. We demonstrate the probe experimentally through *in vivo* experiments in optogenetic mice sensitive to either blue or red photostimulation. A conceptual overview is shown in Fig. 1. Compared with prior reports of dual-color photonic neural probes^17^^,^^19^^,^^20^^,^^23^ (i.e., devices that use integrated waveguides to guide light into the brain for targeted photostimulation), the neural probes demonstrated herein achieve a record number of emitters per shank. Furthermore, this work highlights the potential of combined spatial and wavelength multiplexing for scaling to high-channel count, high-density dual-color photonic neural probes.

## 2 Results

### 2.1 Neural Probe Design and Characterization

**Fig. 2:**
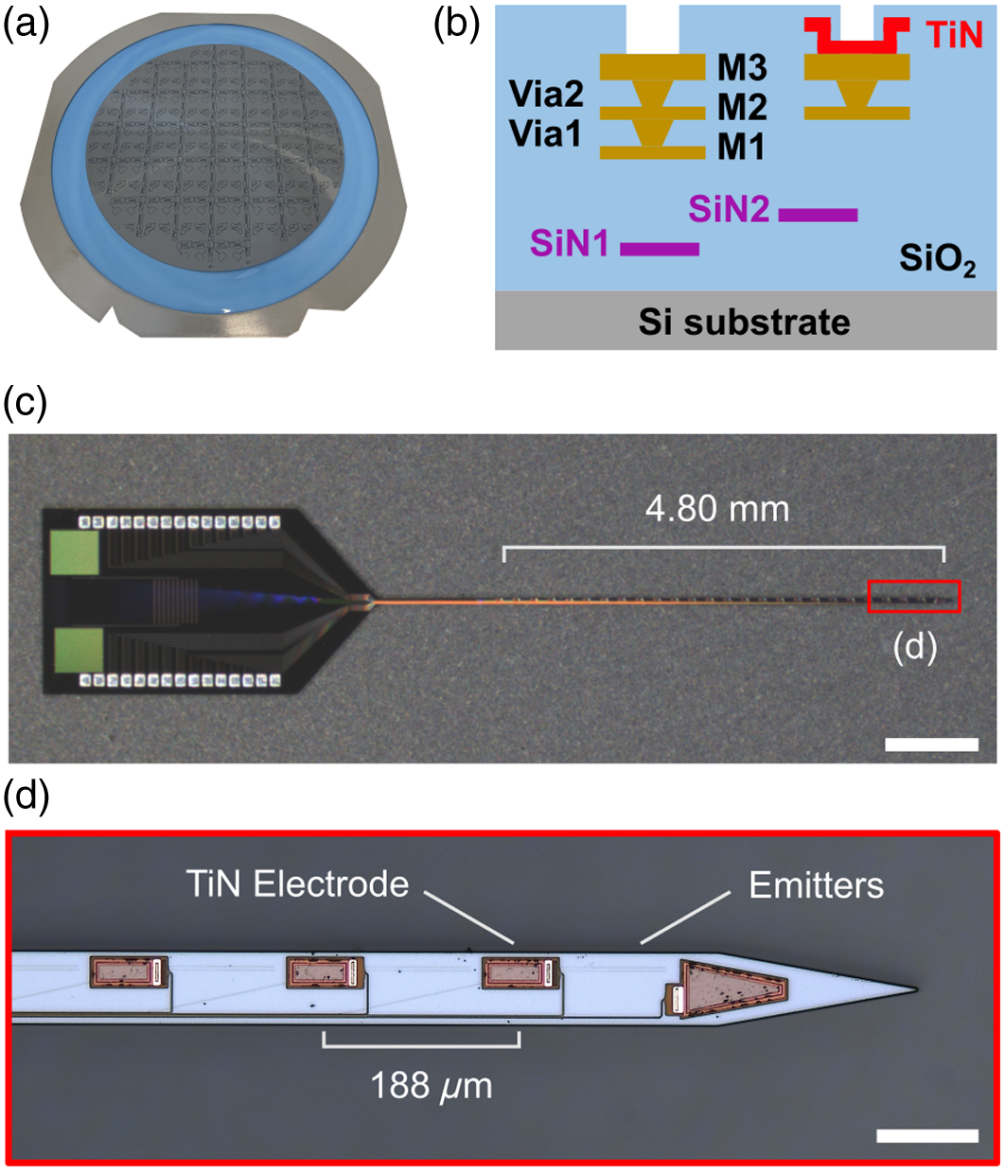
(a) Foundry-fabricated, 200-mm diameter, Si photonic neural probe wafer. (b) Cross-sectional schematic of the neural probes. (c) Micrograph of a fabricated dual-color nanophotonic neural probe. Scale bar: 1 mm. (d) Enhanced micrograph showing TiN electrodes and emitters on the neural probe shank. Scale bar: 100  μm.

Neural probes were designed and simulated using Lumerical finite-difference time-domain optical simulations (Ansys, Inc., Canonsburg, Pennsylvania, United States). Final designs were fabricated by Advanced Micro Foundry (AMF) using a custom visible-light SiN photonic process.^27^ Wafers were received from AMF [Fig. 2(a)], and chips were removed for testing and further processing. The wafer cross-section consisted of two SiN layers (SiN1 and SiN2) with ${\mathrm{SiO}}_{2}$ cladding for photonic routing and three aluminum (Al) metal layers for recording electrophysiological activity [Fig. 2(b)]. Recording electrodes were implemented by coating the top aluminum layer with titanium nitride (TiN). For the neural probes in this study, we aimed to design a probe with sufficiently long span and emitter density to interrogate superficial and deep neural targets with blue (473 nm) and red (638 nm) light. The neural probes, shown in Fig. 2(c), were designed with a 6.09-mm-long and 70-μm-wide shank. Twenty-five TiN recording electrodes (46.5  μm×14  μm), with one additional TiN reference electrode at the shank tip, were distributed linearly along the shank with a 188-μm pitch and span of 4.80 mm [Fig. 2(d)]. An array of 26 fiber-to-chip bilayer edge couplers^28^ was fabricated at the base of the device for coupling light from external lasers using a multicore fiber. To achieve high-density emitters on the shank, our design implemented a wavelength multiplexing scheme whereby each edge coupler interfaced with a nanophotonic waveguide capable of routing both 473- and 638-nm light down the shank of the probe. To reduce waveguide crosstalk, neighboring single-mode waveguides were adiabatically transitioned to dissimilar (detuned) multimode widths of 600 and 700 nm.^29^ On the shank, each routing waveguide terminates with an evanescent directional coupler wavelength demultiplexer designed to filter 473- and 638-nm light to wavelength-specific bilayer grating coupler emitters, consisting of a fully etched grating in SiN2 and a corrugated grating^30^ in SiN1 (Fig. S1 in the Supplementary Material). Overall, each neural probe has 26 routing waveguides that route red and blue light to 26 pairs of grating coupler emitters, for a total of 52 grating coupler emitters. The pitch and span of the grating coupler emitter pairs were 188  μm and 4.82 mm, respectively.

**Fig. 3:**
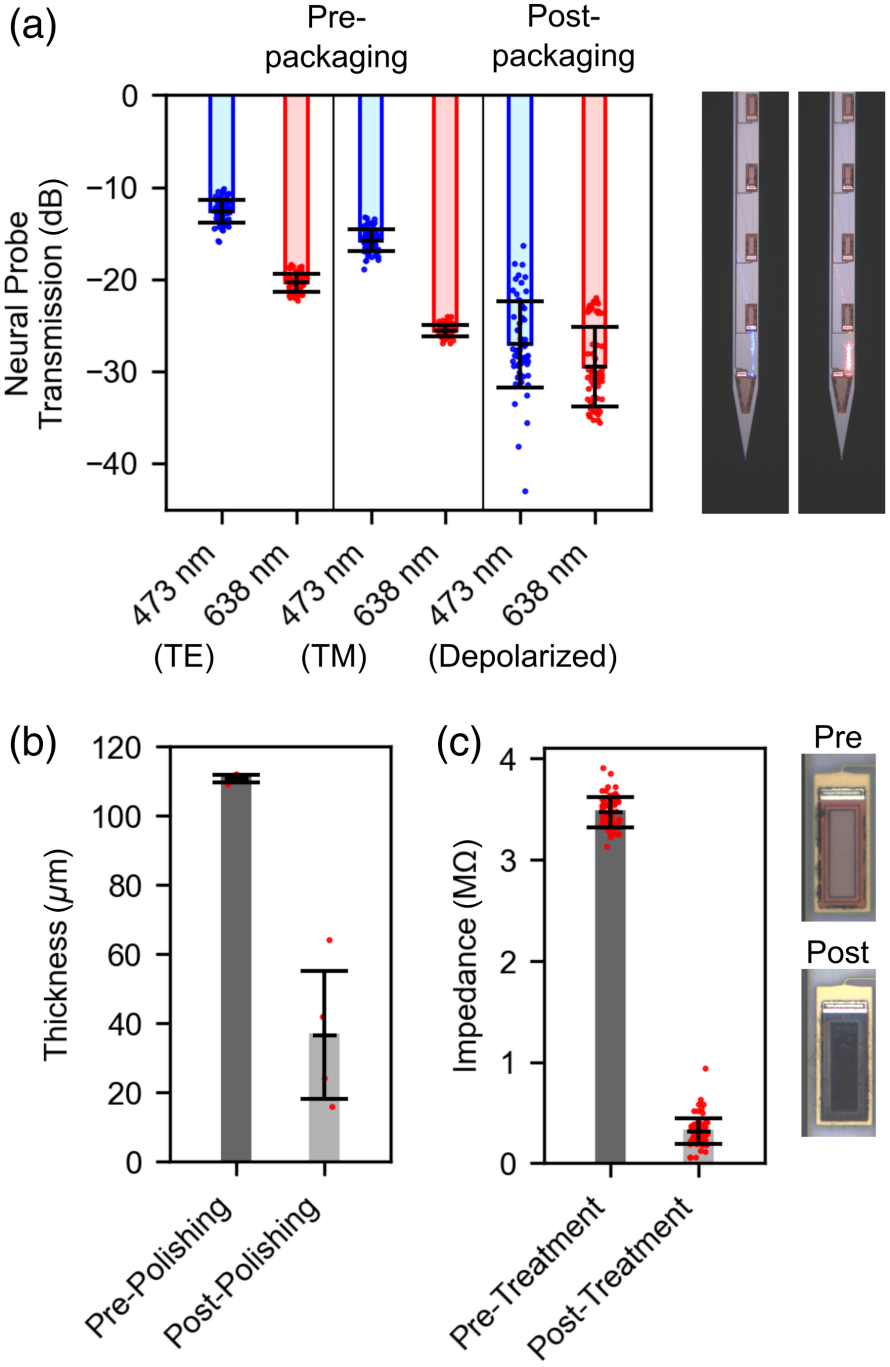
(a) Neural probe transmission measurements for blue (473 nm) and red (638 nm) light before and after packaging (pre-packaging TE and TM measurements: n=78 emitter pairs from three probes; post-packaging depolarized measurements: n=64 emitter pairs from four probes). Micrographs show blue and red lights emitting from the deepest emitter pair on the neural probe. (b) Neural probe shank thickness before and after mechanical polishing (n=4 probes). (c) Electrode impedance magnitudes before and after laser-induced impedance reduction. Micrographs show sample electrodes before and after laser surface roughening (pre-treatment: n=75 electrodes from three probes; post-treatment: n=90 electrodes from four probes).

The optical transmission of neural probe samples prior to packaging (n=78 emitter pairs from three probes) and after packaging (n=64 emitter pairs from four probes) were measured and shown in Fig. 3(a) (left). We define probe transmission as the ratio of output power from a grating coupler emitter to the input power incident at the edge coupler facet. Prior to packaging, the average probe transmission was measured as −12.6±1.2  dB (mean ± SD) and −20.4±1.0  dB for 473- and 638-nm transverse electric (TE)-polarized light and −15.8±1.2  dB and −25.6±0.6  dB for 473- and 638-nm transverse magnetic (TM)-polarized light, respectively. After packaging, transmission was measured using depolarized light (equal parts TE and TM on-chip) to mimic the conditions of the final experiments (see Sec. 4.7), wherein temperature- and stress-induced polarization fluctuations of the multicore fiber coupled to the probe were avoided via depolarized input light. The average transmission for the packaged probes was measured as −27.1±4.7  dB and −29.5±4.3  dB for 473- and 638-nm light, respectively. A micrograph showing 473- and 638-nm light emitting from the deepest pair of grating coupler emitters can be seen in Fig. 3(a) (right). A detailed breakdown of the average transmission data for the nanophotonic probe is presented in Table S1 in the Supplementary Material.

Neural probes were mechanically polished to reduce the overall device thickness in an effort to reduce tissue insertion damage during *in vivo* experiments. The thickness of select neural probe samples before and after mechanical polishing is shown in Fig. 3(b). Prior to mechanical polishing, the thickness of the extracted neural probe chips was 111±1  μm (n=4). After mechanical polishing, the average thickness of the neural probes was reduced to 37±18  μm (n=4).

The electrochemical impedance of the recording electrodes was reduced to improve electrophysiological signal recording during *in vivo* experiments. Electrochemical impedance reduction was achieved using selective femtosecond laser scanning with a two-photon microscope to roughen the electrode surfaces, as described in Sec. 4.2 and our previous work in Ref. 18. The results of the impedance reduction are shown in Fig. 3(c) (left). The impedance magnitude of the untreated TiN electrodes was measured as 3.47±0.15  MΩ (n=75 electrodes from three probes). After impedance reduction, the impedance magnitude of the TiN electrodes was measured as 315±125  kΩ (n=90 electrodes from four probes). Representative images showing the TiN electrodes before and after laser impedance reduction are shown in Fig. 3(c) (right).

### 2.2 On-Shank Directional-Coupler Wavelength-Demultiplexer Performance and Grating Coupler Emitter Beam Profiles

**Fig. 4:**
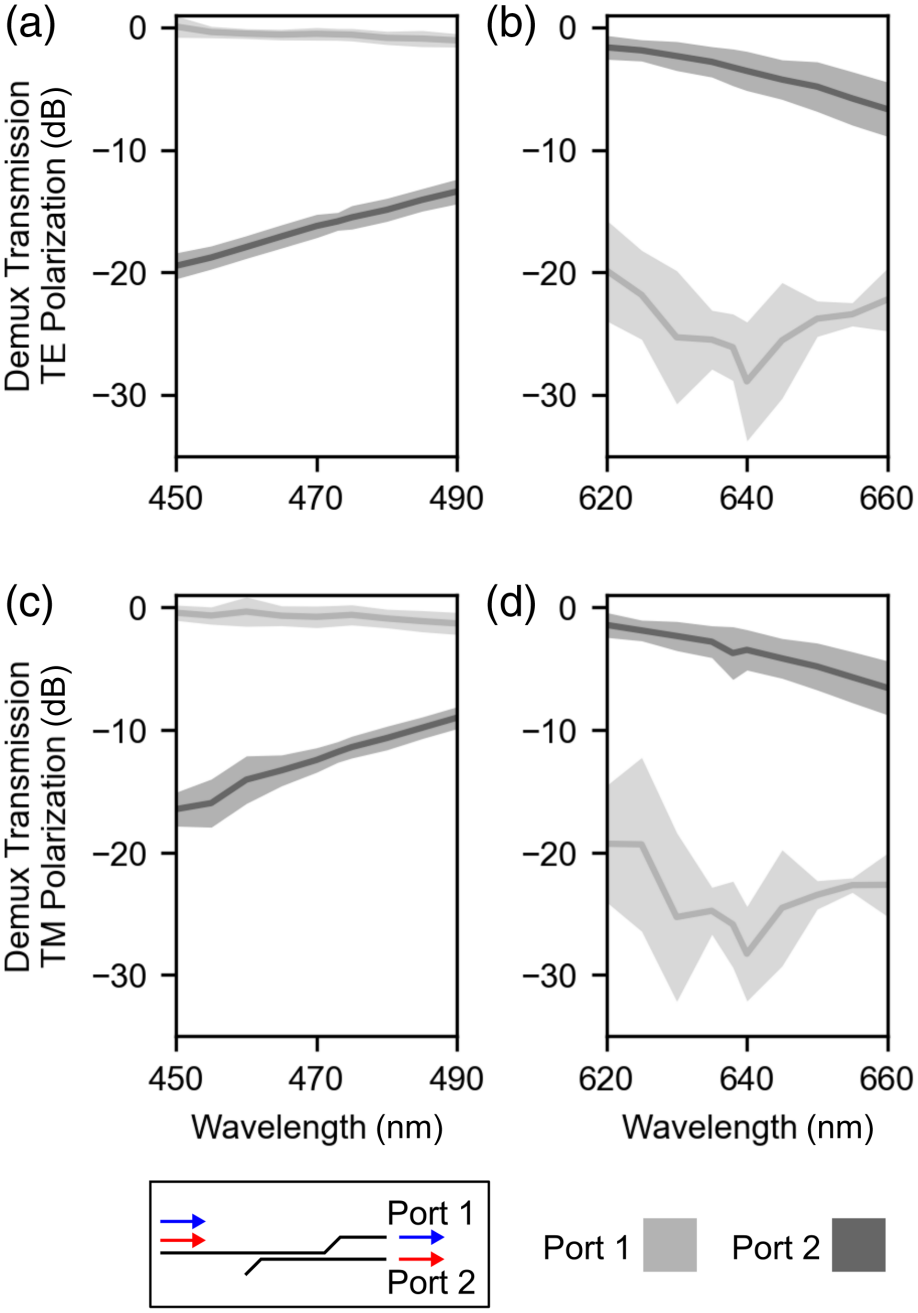
Characterization of wavelength demultiplexer test devices (n=5). For all panels, port 1 was designed to selectively transmit 473-nm light, and port 2 was designed to selectively transmit 638-nm light. (a) and (b) Transmission measurements for TE-polarized light over wavelength spans of (a) 450 to 490 nm and (b) 620 to 660 nm. (c) and (d) Transmission measurements for TM-polarized light for wavelengths (c) 450 to 490 nm and (d) 620 to 660 nm.

Directional coupler demultiplexer (demux) test structures (n=5) were measured to evaluate how well 473- and 638-nm light were coupled to the appropriately designed grating coupler emitters (Fig. 4). Demux test structures were measured using both TE- and TM-polarized light. For each quadrant of Fig. 4, port 1 represents the output port designed to pass 473-nm light, and port 2 represents the output port designed to pass 638-nm light. For TE-polarized light [Figs. 4(a) and 4(b)], the transmission of port 1 was measured as −0.6±0.5  dB and −26.1±2.7  dB for 473- and 638-nm light, respectively, whereas the transmission of port 2 was measured as −15.8±0.7  dB and −3.3±1.5  dB for 473- and 638-nm light, respectively. On average, for TE-polarized light, the extinction ratio among the ports at 473 nm was 15.2 dB, whereas the extinction ratio at 638 nm was 22.8 dB. Similarly, for TM-polarized light [Figs. 4(c) and 4(d)], the transmission of port 1 (2) was measured as −0.7±0.8  dB (−11.8±0.8  dB) and −25.9±3.5  dB (−3.7±2.1  dB) for 473- and 638-nm light, respectively. On average, for TM-polarized light, the extinction ratio at 473 nm was 11.1 dB, whereas the extinction ratio at 638 nm was 22.2 dB.

**Fig. 5:**
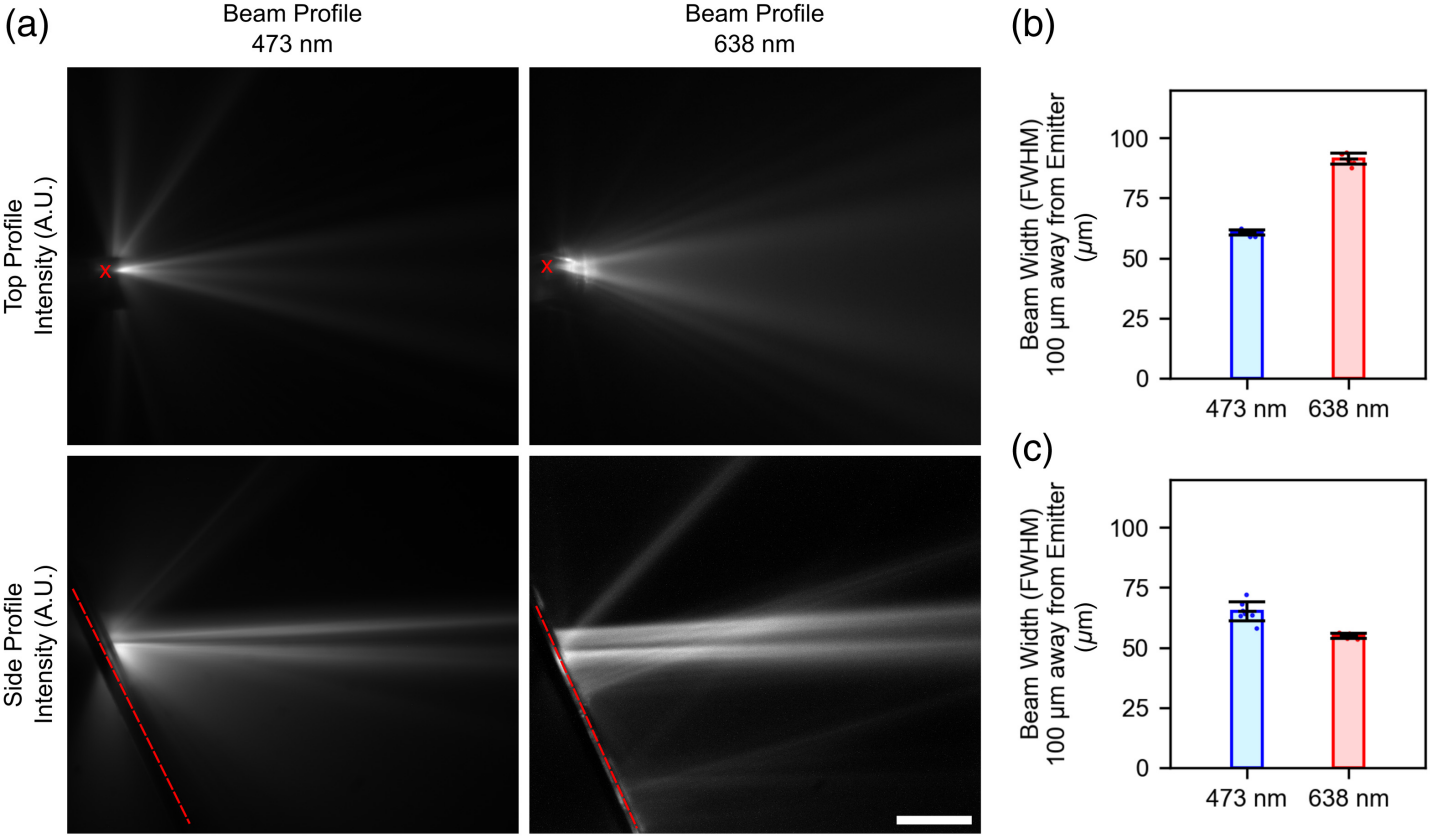
Beam profile measurements for blue (473 nm) and red (638 nm) grating coupler emitters. (a) Representative normalized images showing the beam profiles for 473-nm light in fluorescein solution (left column) and 638-nm light in Alexa Fluor 647 solution (right column). The in-plane/top profile of the beams is shown in the top row, and the side profile of the beams is shown in the bottom row. The neural probe shank is marked with a red x (red dashed line) in the top profile (side profile). Scale bar: 100  μm. (b) FWHM beam width measurements for the 473- and 638-nm beam top profiles measured at 100  μm away from the grating coupler (n=7). (c) FWHM beam width measurements for the 473- and 638-nm beam side profiles measured at 100  μm away from the grating coupler (n=7).

To assess the region of excitation achieved by the blue and red grating coupler emitters, the grating coupler beam profiles were measured in fluorescent solution (Sec. 4.6), and the in-plane and side profiles of the beams were evaluated. Similar to the nanophotonic neural probe transmission measurements, depolarized light was coupled into the neural probes to mimic the conditions of the final experiments (see Sec. 4.7). Representative micrographs of the in-plane/top profiles of the 473- and 638-nm beams are shown in Fig. 5(a) (top row), whereas representative micrographs of the side profiles of the 473- and 638-nm beams are shown in Fig. 5(a) (bottom row). Both the 473- and 638-nm beam profiles achieve a higher fill factor in the side profile by emitting two distinct lobes, emitted from the separate grating structures in SiN2 and SiN1 (Fig. S1 in the Supplementary Material). The full width at half maximum (FWHM) beam widths were measured at a distance 100  μm away from the grating for the top and side profiles (n=7). The beam widths of the top profile were measured as 61±1 μm and 91±2  μm for the 473- and 638-nm beams, respectively [Fig. 5(b)]. Similarly, the measured beam widths of the side profiles were 65±4  μm and 54±1  μm for the 473- and 638-nm beams, respectively [Fig. 5(c)]. The blue emitter also emitted a weak side lobe from the side of the grating structure [see Fig. 5(a), top left]. This side lobe was a characteristic of the TM-polarized component of the output beam and may be avoided in future designs using light-blocking structures (e.g., metal wiring layers) on the side of the grating.

To estimate the beam widths in tissue, we simulated the emitter beam profiles in scattering media using the beam propagation method (see Fig. S3 in the Supplementary Material), as outlined in Refs. 31 and 32, with scattering parameters derived from previous literature.^33^^,^^34^ To model the depolarized input to the neural probe, TE- and TM-polarization propagation simulations were performed independently and combined using an incoherent sum, with weights derived from experimental measurements of the TE and TM transmission of the probe. After propagating, beams were rotated to match the orientation of the experimentally measured beam profiles [Fig. S3(a), top row, in the Supplementary Material]. Based on this analysis, for gratings simulated with the designed dimensions in Sec. 4.1, the beam widths 100  μm away from the gratings were calculated as 61  μm (473-nm beam, side profile), 72  μm (473-nm beam, top profile), 35  μm (638-nm beam, side profile), and 51  μm (638-nm beam, top profile) [Fig. S3(b), left, in the Supplementary Material]. We observed that the simulated beams had narrower widths than expected from measurements, due to greater emission from the SiN2 grating than the SiN1 grating. Cross-sectional transmission electron microscopy was performed on one probe sample to compare the designed and fabricated waveguide thicknesses. The SiN thicknesses were within 3% to 7% of the designed values; however, the gap between the SiN1 and SiN2 layers (interlayer ${\mathrm{SiO}}_{2}$ thickness) was significantly larger than expected (162 versus the 100 nm designed value). With these updated dimensions, we explored whether these modified dimensions would explain the differences we observed between the simulated and experimentally measured beams. Additional beam propagation simulations in scattering media [Fig. S3(a), bottom row, in the Supplementary Material] yielded beam widths 100  μm away from the gratings of 55  μm (473-nm beam, side profile), 34  μm (473-nm beam, top profile), 51  μm (638-nm beam, side profile), and 50  μm (638-nm beam, top profile) [Fig. S3(b), right, in the Supplementary Material]. Overall, these simulated results still appeared to underestimate the measured beam widths, possibly due to differences between the simulated and fabricated grating dimensions (and resulting grating strengths).

### 2.3 *In Vivo* Evaluation with Dual-Color Photostimulation and Electrophysiological Recording

To evaluate the performance of the neural probes, we conducted experiments in optogenetic mice responsive to either blue or red photostimulation. A nanophotonic neural probe was connected to a custom-designed dual-color laser scanning system (Fig. S2 in the Supplementary Material) via a 16-core multicore optical fiber^35^ (see Sec. 4.7) for delivering programmatically defined high-intensity blue and red light pulse trains (10 pulse trains, 10 pulses per pulse train, 30 ms pulse width, and 5 Hz) through up to 16 of the available 26 emitter pairs. The nanophotonic probe was implanted along with a Neuropixels 2.0 single-shank probe^25^ to capture high-density electrophysiological data from the target brain region during dual-color photostimulation at multiple depths.

**Fig. 6:**
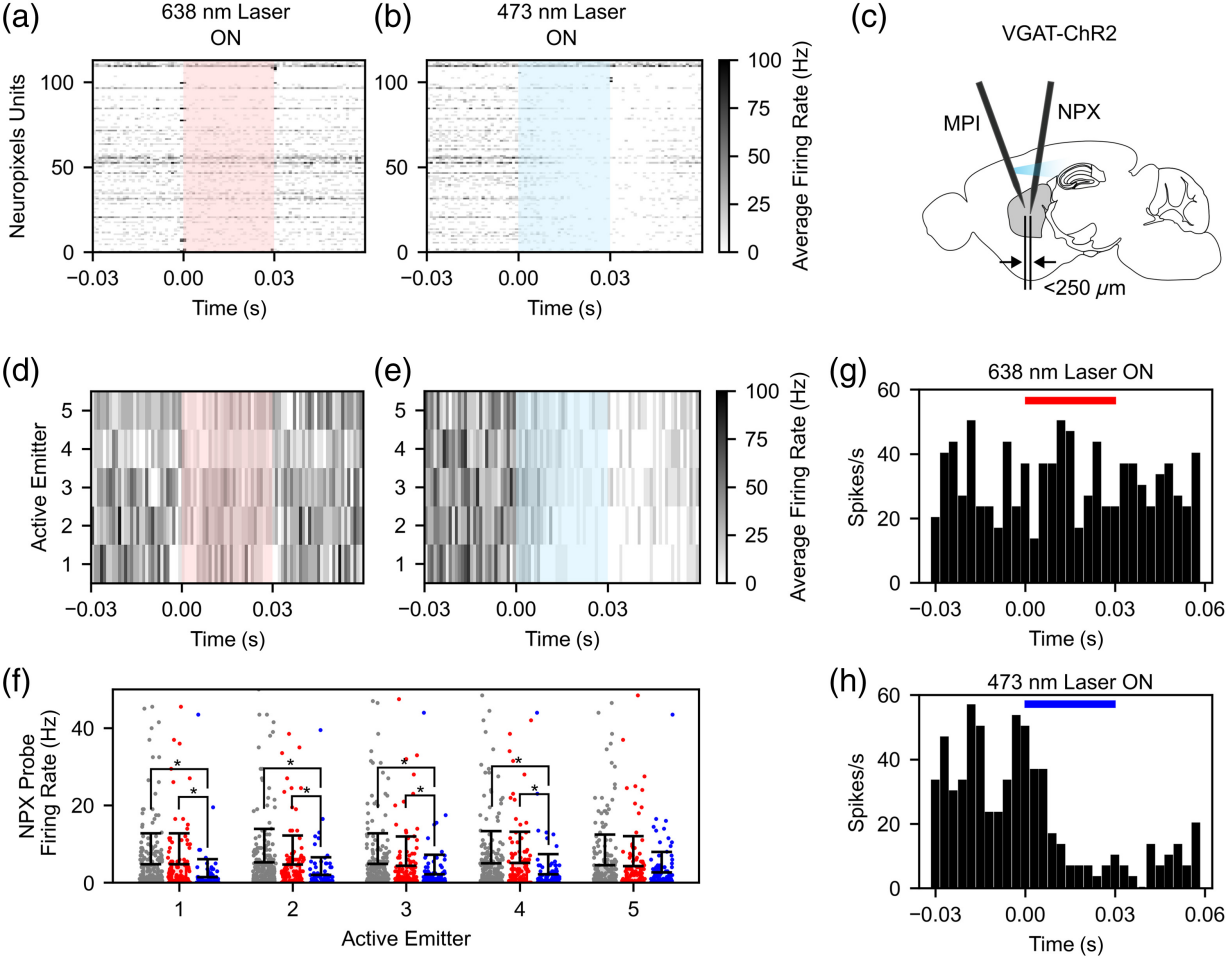
*In vivo* photostimulation experiment results in the cortex of a VGAT-ChR2 mouse. (a) and (b) Representative spike-sorted data of Neuropixels units (n=113) during (a) red and (b) blue photostimulation. (c) Conceptual image showing the relative position of the nanophotonic (MPI) and Neuropixels (NPX) probes during the experiment. See Fig. S4 in the Supplementary Material for more detailed information. (d) and (e) Representative data from one Neuropixels unit during (d) red and (e) blue photostimulation from emitters 1 to 5. (f) Average firing rate data for Neuropixels units before and during photostimulation from emitters 1 to 5. The colors of the bars in panel (f) correspond to the color of photostimulation [left: gray (firing rate before photostimulation); middle bar: red; right bar: blue]. (g) and (h) Representative average peristimulus time histograms for one unit during (g) red and (h) blue photostimulation.

We assessed the functionality of blue photostimulation in the cortex of a blue light–sensitive VGAT-ChR2 mouse (Fig. 6), which expresses Channelrhodopsin-2 in all GABAergic (inhibitory) interneurons. To maximize the blue photostimulation intensity, the end of the multicore fiber was moved into the blue laser focal position in the dual-color laser scanning system [Fig. S2(d) in the Supplementary Material]. At this position, the total system insertion loss was measured as 34.0±1.0  dB (41.7±0.7  dB) at 473 (638) nm, for an average maximum emitter power output of 119.4  μW and 12.2  μW for 473- and 638-nm light, respectively. Blue light pulse trains were delivered to the cortex through emitters 1 (deep) to 5 (superficial), while electrophysiological data from the nanophotonic probe were monitored. Red photostimulation was also delivered as a control for the blue photostimulation, as the relative sensitivity of ChR2 to red light is ≪1% that of blue light.^36^ Electrophysiological data from the nanophotonic neural probe underwent spike sorting (Sec. 4.9); however, no high-quality units were detected. In addition to the low-density electrophysiological recordings captured by the nanophotonic probe, high-density electrophysiological recordings were captured using a Neuropixels probe placed ∼250  μm away from the tip of the nanophotonic probe (see Fig. S4 in the Supplementary Material for additional details). Representative data showing the average firing rate of all high-quality units (n=113) detected by the Neuropixels probe in response to photostimulation from emitter 3 are shown in Figs. 6(a) and 6(b). During red photostimulation, no obvious effects on the firing rate were observed [Fig. 6(a)]. In contrast, widespread reductions in firing rate were observed during blue photostimulation, consistent with synaptically driven inhibition of the local population by blue light–sensitive GABAergic interneurons [Fig. 6(b)]. A conceptual image showing the relative positions of the nanophotonic probe and Neuropixels probe is shown in Fig. 6(c). Representative data showing the average firing rate of one unit in response to red and blue photostimulation from each emitter can be seen in Figs. 6(d) and 6(e). Here, broad inhibition is seen for blue photostimulation from all emitters. The average firing rate of all Neuropixels units was quantified before and during photostimulation through emitters 1 to 5 for comparison [Fig. 6(f): average firing rate before photostimulation from emitters 1 to 5: 4.8±8.0, 5.3±8.6, 4.8±7.9, 5.0±8.3, and 4.5±7.9  Hz; average firing rate during red photostimulation from emitters 1 to 5: 4.7±8.0, 4.7±7.5, 4.4±7.6, 5.1±8.1, and 4.3±7.7  Hz; average firing rate during blue photostimulation from emitters 1 to 5: 1.4±4.6, 2.0±4.6, 2.2±5.0, 2.2±5.2, and 2.6±5.3  Hz]. For nearly all emitters, the average firing rate during blue photostimulation was significantly lower than both the firing rate before photostimulation (emitters 1, 2, 3, and 4: p<0.001; emitter 5: p=0.071, Mann–Whitney U rank test) and the firing rate during red photostimulation (emitters 1, 2, and 4: p<0.001; emitter 3: p=0.003; emitter 5: p=0.088, Mann–Whitney U rank test). Lastly, representative average peristimulus time histograms for one unit across all trials are shown in Figs. 6(g) and 6(h) to highlight the changes in firing rate during red and blue photostimulation, respectively.

To determine whether the observed reduction in firing rate was due to activation of inhibitory interneurons in the VGAT-ChR2 mouse or due to thermal effects, we performed another experiment in a wild-type mouse with blue photostimulation. Here, a Neuropixels 2.0 probe was used to record neural activity in the cortex during comparable blue photostimulation pulse trains (30-ms pulse width, 5 Hz) from a collimated 473-nm laser beam at laser powers of 5, 10, and 20 mW (with intensities of 6.4, 12.8, and $25.6\;\mathrm{mW}/{\mathrm{mm}}^{2}$, respectively). Across all trials, no reduction in firing rate was observed during these experiments (Fig. S5 in the Supplementary Material). Furthermore, we simulated the heating effects resulting from the maximum pulse train settings used in our nanophotonic probe experiment (119.4  μW, 30-ms pulse width, 5 Hz) and found that tissue temperatures rise by less than 0.15°C (Fig. S6 in the Supplementary Material).

Following the experiments, we attempted to modify the photostimulation pulse train settings for higher frequency (10-Hz pulse frequency and 10-ms pulse width). However, for the blue laser source, these settings resulted in irreparable damage to the selected emitters (1 and 2), likely due to light-induced damage to the UV-curing epoxy which bonded the multicore fiber to the probe. We investigated this damage mechanism by measuring the time-to-failure (due to blue 445-nm laser light exposure) of two cleaved single-mode fibers bonded with the same UV-curing epoxy used with the neural probes. The fibers had similar mode field diameters to our multicore fibers^35^ (approximating the intensities at the input facet of the neural probes), and blue laser light at various power levels was transmitted through the epoxied fiber junction (Fig. S7 in the Supplementary Material, n=3). We observed an exponential relationship between the input laser power and the time-to-failure [Fig. S7(a) in the Supplementary Material]—a point at which the transmitted power suddenly and irreversibly decreased to nearly zero [Fig. S7(b) in the Supplementary Material]. At continuous laser powers of >30  mW, the bonded junction could operate for tens of seconds before failing. At lower continuous laser powers ≤20  mW, the junction could operate for several minutes before failing. For our *in vivo* experiment in the VGAT-ChR2 mouse, the estimated average laser power emitted by the MCF at the neural probe chip facet was 16.2 mW, corresponding to a peak power of 107.7 mW [Fig. S2(d) in the Supplementary Material] and duty cycle of 15%, with 170 ms between each pulse for heat dissipation.

**Fig. 7:**
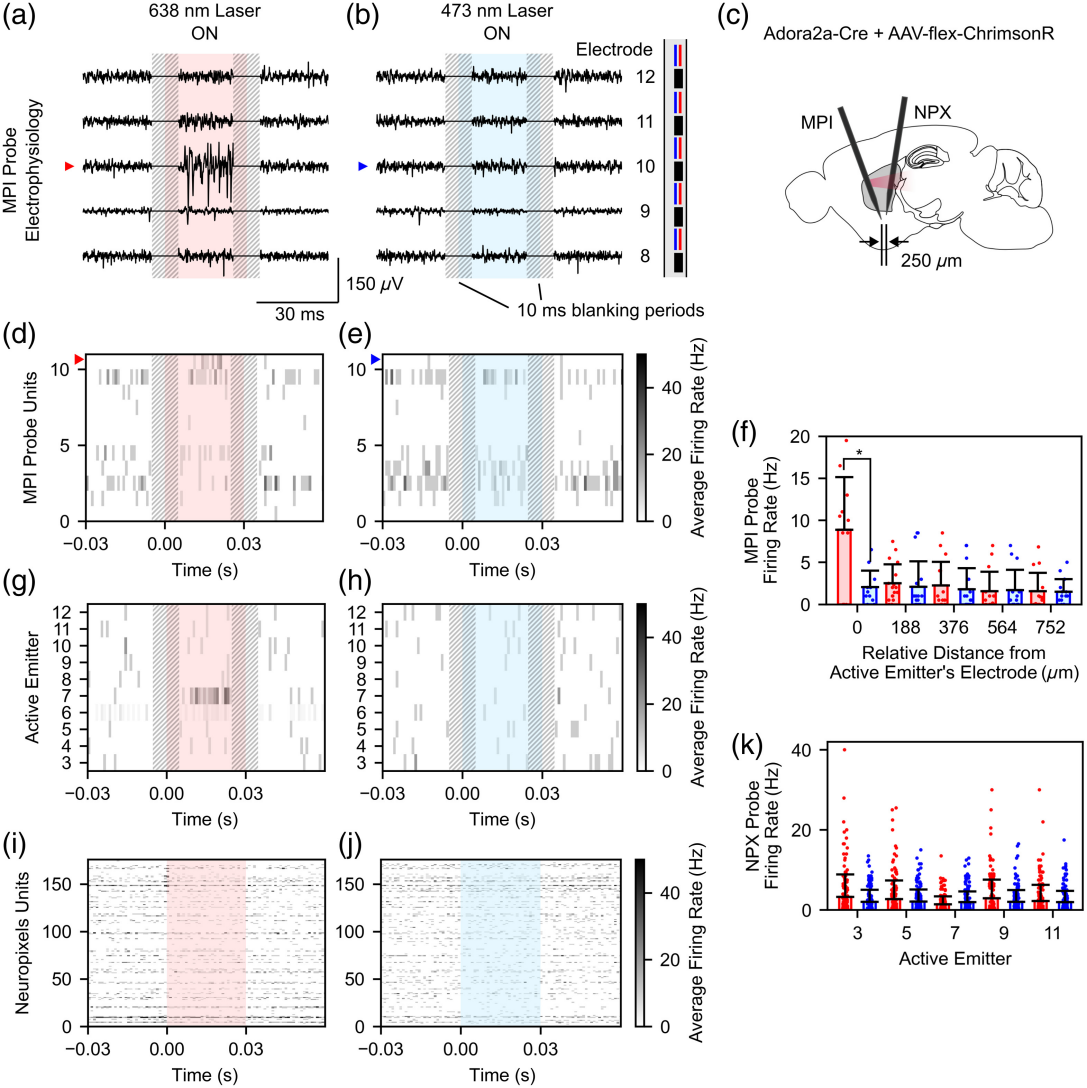
*In vivo* photostimulation experiment results in the striatum of an Adora2a-Cre + AAV-flex-ChrimsonR mouse. (a) and (b) Representative preprocessed electrophysiological data from electrodes 8 to 12 on the nanophotonic probe during (a) red and (b) blue photostimulation. A 10-ms blanking period, centered on each pulse onset and offset, was applied for each stimulation pulse to remove stimulation artifacts. (c) Conceptual image showing the relative position of the nanophotonic (MPI) and Neuropixels (NPX) probes during the experiment (Fig. S4 in the Supplementary Material). (d) and (e) Representative spike-sorted data of nanophotonic probe units (n=11) during (d) red and (e) blue photostimulation for one active emitter. (f) Average binned firing rate data for nanophotonic probe units as a function of the distance from the active emitter. (g) and (h) Representative firing data from one nanophotonic probe unit during (g) red and (h) blue photostimulation from emitters 3 through 12. (i) and (j) Representative spike-sorted data of Neuropixels units (n=176) during (i) red and (j) blue photostimulation. (k) Average firing rate data for Neuropixels units for photostimulation from emitters 3, 5, 7, 9, and 11. The color of the bars in panels (f) and (k) corresponds to the photostimulation color (left bar: red; right bar: blue).

Next, we assessed the functionality of red photostimulation in a mouse that expresses red-light-sensitive ChrimsonR in indirect medium spiny neurons (MSN) of the striatum (Adora2a-Cre mouse injected with AAV-flex-ChrimsonR). To maximize the red photostimulation intensity, the end of the multicore fiber was moved into the red laser focal position in the dual-color laser scanning system [Fig. S2(d) in the Supplementary Material]. At this position, the total system insertion loss was measured as 43.3±0.5  dB (38.3±0.7  dB) at 473 (638) nm, for an average maximum emitter power output of 14.0 and 26.6  μW for 473- and 638-nm light, respectively. Red photostimulation pulse trains were delivered to the dorsal striatum and overlying cortex through emitters 3 (deep) to 12 (superficial), whereas evoked electrophysiological activity was measured by the nanophotonic probe and a Neuropixels probe (approximate distance between probe tips: 250  μm, see Fig. S4 in the Supplementary Material for more detailed information). Blue photostimulation was delivered as a control, with an understanding that high-intensity blue light may still stopped here but with a lower yield.^9^ Representative images of the preprocessed electrophysiological data (see Sec. 4.9 for details on preprocessing) evoked during red and blue photostimulation from emitter 10 are shown in Figs. 7(a) and 7(b). A conceptual image showing the relative positions of the nanophotonic probe and Neuropixels probe is shown in Fig. 7(c). For red photostimulation, large amplitude spikes were observed on the recording electrodes nearest to the active emitter. This localized activity was not observed during blue photostimulation. Representative average firing rate data of spike-sorted units during photostimulation from emitter 10 can be seen in Figs. 7(d) and 7(e). The approximate level of the active emitter is represented using a red or blue arrow. As shown in the preprocessed electrophysiological data, localized activity was observed during red photostimulation [Fig. 7(d)] but not blue photostimulation [Fig. 7(e)]. We quantified this local activation by calculating the average firing rate of all units (n=11) as a function of the unit distance from the active emitter for photostimulation from emitters 3 to 12 [Fig. 7(f): average firing rate during red photostimulation of units increasingly distant from the active emitter: 8.9±6.3, 2.5±2.2, 2.2±2.8, 1.6±2.3, and 1.6±2.2  Hz; average firing rate during blue photostimulation of units increasingly distant from the active emitter: 2.1±2.0 , 2.1±3.0, 1.8±2.5, 1.7±2.4, and 1.5±1.5  Hz]. Units nearest to the active emitter had significantly higher firing rates for red photostimulation when compared with blue photostimulation (p=0.047, Mann–Whitney U rank test). Firing rate data from one representative unit in response to photostimulation from emitters 3 to 12 is shown in Figs. 7(g) and 7(h). Again, we observed highly localized activation near one emitter for red photostimulation but not blue photostimulation. In addition to the nanophotonic probe neural recordings, electrophysiological data were measured further away from the neural probe using a Neuropixels 2.0 probe. Representative average firing rate data for the Neuropixels units (n=176) during red and blue photostimulation from emitter 10 can be seen in Figs. 7(i) and 7(j). No clear differences were observed in the firing rate of any units during red and blue photostimulation. The average firing rate for all units was calculated during photostimulation from emitters 3, 5, 7, 9, and 11 (representing a photostimulation span of 1.5 mm) to quantify the regional effects of photostimulation [Fig. 7(k): average firing rate during red photostimulation from emitters 3, 5, 7, 9, and 11: 3.3±5.6, 2.7±4.7, 1.4±2.0, 2.9±4.7, and 2.2±4.0  Hz, respectively; average firing rate during blue photostimulation from emitters 3, 5, 7, 9, and 11: 2.0±3.0, 2.1±3.0, 1.9±2.7, 2.0±3.0, and 1.9±2.8  Hz, respectively]. Overall, no differences in the average firing rate of units during red and blue photostimulation were observed, and potential reasons for this are discussed in Sec. 3. In comparison with the broad inhibition seen on the Neuropixels probe in the VGAT-ChR2 mouse, the lack of any widespread response to red light in this experiment could be due to the lower maximum light power from the red versus blue emitters, or the more limited spread of the virus used to express ChrimsonR.

## 3 Discussion and Conclusion

We have characterized and demonstrated the functionality of a nanophotonic neural probe design with combined spatial multiplexing and on-shank wavelength-demultiplexers for high-density dual-color photostimulation and electrophysiological recording in two separate experimental settings: in a blue-light sensitive VGAT-ChR2 mouse and in a red-light sensitive Adora2a-Cre + AAV-flex-ChrimsonR mouse. In the current configuration, we connected 16 of the total 26 emitter pairs to a laser scanning system via a custom 16-core multicore fiber for red and blue photostimulation across a span of 2.92 mm—sufficient for stimulating and recording from cortex as well as deep brain structures in the mouse. Even in this limited configuration, this represents, to the authors’ knowledge, the most addressable emitters on a single photonic neural probe shank to date when compared with other dual-color devices using integrated waveguides to stimulate neural tissue.^17^^,^^19^^,^^20^^,^^23^ Although the emitter density of the current device lags behind the emitter densities achieved by state-of-the-art single- and dual-color optoelectronic probes,^13^^,^^37^ this work advances the emitter densities achievable with integrated photonics–based neural probes. A summary of our design in comparison with other state-of-the-art photonic and optoelectronic probe designs is shown in Table 1. With the combination of two multicore fibers, all 26 emitter pairs could be controlled, and the full 4.80-mm emitter span could be realized, which would improve this metric even further. We envision such high-density and long-span neural probes supporting optogenetic studies involving deep neural structures or neural systems spanning several millimeters, such as in the cortex and deep brain structures such as the basal ganglia,^38^^,^^39^ hippocampus,^40^ or spinal cord.^41^ Because our nanophotonic neural probes are fabricated in a commercial Si photonics foundry with minimal post-processing, a direct path exists for scaling fabrication volumes to broadly disseminate these devices within the neuroscience community.

**Table 1:** Comparison of state-of-the-art photonic and optoelectronic neural probes for multicolor photostimulation and electrophysiological recording.

Referenced work	Refs. 19 and 20	Ref. 17	Ref. 12	Ref. 13	This work
Architecture	Photonic (SiN)	Photonic (SiN)	Optoelectronic (μLED)	Optoelectronic (OLED)	Photonic (SiN)
Emitter power	100 μW (blue)	5 to 10 μW (blue and red)	∼200 nW (blue, red)	100 nW (blue)	212 μW (blue)
	372 μW (red)			40 nW (orange)	88 μW (red)
Total emitters	14 × 2 colors	3 × 2 colors	16 × 2 colors	1024 (orange or blue)	26 × 2 colors
Emitters/shank	14 × 2 colors	3 × 2 colors	16 × 2 colors	256 (orange or blue)	26 × 2 colors
Total electrodes	960 (384 selectable)	64	17	32 (bonded multielectrode array)	26
Electrodes/shank	960 (384 selectable)	64	17	32 (bonded multielectrode array)	26
Shank dimensions	10 mm×70 μm×33 μm	1 mm×45 μm×15 μm	5 mm×200 μm×50 μm	6.17 mm×100 μm×55 μm	6.10 mm×70 μm×37 μm
Wavelengths	450 and 638 nm	450 and 655 nm (central wavelengths)	462 and 625 nm	500 and 616 nm (central wavelengths)	473 and 638 nm

We have shown that evanescent directional-coupler wavelength demultiplexers provide an effective and compact method for demultiplexing two spectrally distinct wavelengths of light (i.e., red and blue) to separate grating emitters on the neural probe shank. With an overall width of ∼3  μm, these devices are considerably more compact than microring resonator demultiplexers, which have diameters from 8 to 14  μm^17^ (limited by waveguide bend losses). This compactness allows for more shank area to be allocated for waveguide routing. Moreover, the relatively broad bandwidth of directional-coupler devices reduces sensitivity to fabrication variations. For the directional-coupler wavelength-demultiplexer presented here, the average extinction ratio among output ports ranged from 11.1 to 22.8 dB, depending on the wavelength and polarization. In the case of the lowest extinction ratio (i.e., for 473-nm TM-polarized light), ∼10% of the input light may transmit to the red light–optimized grating coupler emitter instead of the blue light–optimized emitter, resulting in a second, lower-intensity beam being emitted by the emitter pair. Due to the close proximity of the red and blue emitters within each emitter pair, photostimulation would still be highly localized to the same stimulation depth; however, the volume of photostimulation would be altered by the combined spatial profile of the two beams. In particular, the red light emitter would emit 473-nm TM-polarized light at an angle of 35 to 40°, broadening the side profile of the beam and resulting in a larger photostimulation volume (data not shown). To avoid this effect, multiple directional-coupler wavelength demultiplexers could be cascaded before the emitter pair to improve the extinction ratio further and reduce emitter crosstalk. In addition, leveraging the broadband transparency of our SiN waveguides,^22^ more advanced demultiplexers with additional wavelength channels may be implemented to extend the probe operation to green and/or yellow wavelengths—for compatibility with a larger set of opsins. This functionality extension would require design and optimization of the demultiplexers for low crosstalk over a denser set of operation wavelengths as well as increased scanning system complexity.

We have demonstrated that high-intensity red and blue photostimulation pulse trains can be delivered with millisecond precision and average output powers of 119.4 and 29.5  μW for blue and red light, respectively, for a particular nanophotonic probe sample *in vivo*. Based on the average transmission of probes measured in this study [see Fig. 3(a)] and the maximum red and blue optical power available from the dual-color scanning system [see Fig. S2(d) in the Supplementary Material], the average maximum output power that could be achieved by the current design is 212  μW for 473-nm light and 88  μW for 638-nm light. These emitter powers are primarily limited by probe transmission, which itself is limited by variability in fiber-to-chip coupling. After packaging, probe transmission decreases by as much as 15 dB due to variable fiber-to-chip coupling, typically related to drift and misalignment of the multicore fiber relative to the on-chip edge couplers during or shortly after UV-curing. To reach the high output powers demonstrated in this study, we compensate for low probe transmission using high-power externally coupled lasers, with output powers ranging from 180 to 300 mW. However, high-intensity laser light may also damage the packaged device. When adjusting the pulse train settings, increasing the frequency of the blue photostimulation pulse train resulted in emitter damage, likely due to damage to the UV-curing epoxy at the base of the nanophotonic probe. Based on our extended analysis investigating the time-to-failure of single-mode fibers bonded with UV-curing epoxy (Fig. S7 in the Supplementary Material), we observed that this failure mode is intrinsically linked to the average laser power transmitted through the epoxy junction. At lower average powers (<20  mW), the epoxy can withstand blue laser light for several minutes without damage. This aligns well with our low-frequency experimental data, where we did not observe emitter failure during blue photostimulation due to the low average laser power at the MCF (time-averaged laser power at MCF: 16.2 mW). Thus, to enable high-frequency blue photostimulation, the average laser power at the MCF output must be limited to ∼15  mW, in addition to having sufficient time among extended laser pulse trains to permit thermal relaxation. Although the prototype neural probes in this work are limited in peak laser power for high-frequency blue pulse trains, future optimization of the neural probe photonic circuitry for increased optical transmission—enabling lower laser powers to deliver greater emitter output powers—is expected to alleviate this constraint. Meanwhile, exploration of alternative epoxies with the goal of improved blue laser light tolerance may further mitigate this limitation.

We have also characterized the emitter beam profiles experimentally (Fig. 5) and in simulation (Figs. S1 and S3 in the Supplementary Material) so that the region of excitation can be modeled in future work. Notably, our simulation results predict smaller beam widths than our experimental measurements in non-scattering media. This discrepancy appears related to a higher estimated grating strength for the SiN1 gratings, and we hypothesize that these differences may be due to fabrication errors related to the grating features or the bottom cladding thickness.

We have demonstrated the functionality of the nanophotonic probe by applying dual-color photostimulation and recording neural activity in two optogenetic mouse experiments. In a blue light–sensitive VGAT-ChR2 mouse, we observed widespread reductions in firing rate during blue photostimulation for neurons recorded by a Neuropixels probe placed ∼250  μm away from the nanophotonic probe [Figs. 6(b), 6(e), 6(f), and 6(h)]. These effects were not observed during red photostimulation [Figs. 6(a), 6(d), 6(f), and 6(g)] or during comparable and higher-intensity blue photostimulation in a wild-type mouse (Fig. S5 in the Supplementary Material), suggesting that they were caused by optogenetic activation of ChR2+ interneurons and not due to thermal effects.^34^^,^^42^ Similarly, our simulation results also suggest that heating caused by photostimulation in our experiments was below 0.15°C (Fig. S6 in the Supplementary Material), which is far lower than temperature changes known to affect neural activity (typically >1°C).^34^^,^^42^ Although red light appears to reach the Neuropixels probe (stimulation artifacts are more clearly observed at the Neuropixels probe during red photostimulation than blue photostimulation, perhaps owing to reduced scattering of red light in tissue), this stimulation does not result in reduced neuronal firing rates due to low interaction of red light with ChR2+ interneurons.^36^ Overall, these results agree well with the expected effects of blue photostimulation in VGAT-ChR2 mice, where the activation of inhibitory interneurons is well known to inhibit cortical neurons millimeters away from the stimulation site, resulting in widespread neuronal inhibition.^43^ In addition, in a red light–sensitive Adora2a-Cre + AAV-flex-ChrimsonR mouse, we observed highly localized excitation during red photostimulation for neurons recorded by the nanophotonic probe [Figs. 7(a), 7(d), 7(f), and 7(g)] but not for neurons recorded by a Neuropixels probe implanted 250  μm away [Figs. 7(i) and 7(k)] or during blue photostimulation [Figs. 7(b), 7(e), 7(f), 7(h), 7(j), and 7(k)].

Compared with the VGAT-ChR2 mouse, these differences in measured neural responses can largely be explained by two factors. First, activation of ChrimsonR+ MSNs within the striatum of the Adora2a-Cre + AAV-flex-ChrimsonR mouse does not lead to widespread inhibition in the local neural network, in stark contrast to photostimulation in the VGAT-ChR2 mouse. Therefore, for these experiments, we must measure the changes in the firing rate of neurons directly affected by photostimulation. This important distinction limits the detection of evoked responses to neurons which both directly receive sufficient photostimulation intensities to evoke spiking ($>1\;\mathrm{mW}/{\mathrm{mm}}^{2}$)^9^^,^^44^ and are within recording distance (∼140  μm)^45^ of either the nanophotonic or Neuropixels probes. Second, based on the average separation distance along the entire length of the nanophotonic and Neuropixels probes in this experiment (∼750  μm, Fig. S4 in the Supplementary Material) and the measured beam widths and beam divergences seen in Fig. 5, we can estimate the average intensity of red photostimulation at the Neuropixels probe to be $0.29\;\mathrm{mW}/{\mathrm{mm}}^{2}$, below levels required to activate ChrimsonR+ neurons. Indeed, the true intensity is likely even lower due to light scattering in tissue. Similarly, we can estimate the intensity of the beam within 140  μm of the nanophotonic probe, which can be estimated as $>1.72\;\mathrm{mW}/{\mathrm{mm}}^{2}$. This intensity is sufficient for activating ChrimsonR+ neurons recorded by the nanophotonic probe, which explains why we detect locally evoked activation during red photostimulation but not regional activation. In addition, if we estimate the recording volume around each nanophotonic probe electrode as a hemisphere with radius 140  μm, and model the red beam geometrically as a wedge with thickness 54  μm [average side-profile beam width, Fig. 5(c)] and divergence of 28° [divergence of top-profile, Fig. 5(a)], we can estimate the volume overlap between photostimulation and neural recording at the nanophotonic probe. As each emitter is located between two electrodes on the nanophotonic probe (one electrode deep relative to the emitter and one electrode superficial relative to the emitter), we complete this estimation separately for both cases. Using this approximation, we estimate that the volume overlap between the active emitter and the nearest deep electrode is 5.3%, whereas the overlap between the active emitter and the nearest superficial electrode is 1.3%. This simple geometrical interpretation supports our observations that evoked activity was preferentially observed on the nearest deep electrode during red photostimulation in the Adora2a-Cre + AAV-flex-ChrimsonR mouse [Fig. 7(f)].

The current probe design is able to record neural data with 26 electrodes across a wide span but with low resolution. In practice, these recordings can offer some insight into the local effects of photostimulation. However, when paired with high-density electrophysiology probes (as demonstrated with Neuropixels probes in the current study), the regional effects of stimulation can also be explored at greater resolution. In combination, tools separately optimized for either high-density photostimulation or high-density electrophysiological recording may offer researchers the ability to probe neural function at previously unachievable resolution and scale.

In future designs, we envision the emitter density of the nanophotonic neural probes improving further by employing a combination of denser waveguide routing, more compact demultiplexing structures, and multiple photonic layers for independent routing. Moreover, using multicore fibers with more than 16 cores, a greater number of addressable photonic channels could be realized. In addition, to transition from acute to chronic experimental configurations, further research is necessary to produce a reliable connector for the multicore fiber connected to the neural probe. In the current configuration, the multicore fiber remains permanently attached to the probe after packaging, limiting compatibility with chronic experimental designs, where the mouse would ideally be disconnected from the external laser scanning system outside of experiments. A reliable multicore fiber connector may permit the system to be disconnected/reconnected, permitting chronic probe implantation. Alternatively, on-chip Mach–Zehnder switching architectures^16^^,^^19^^,^^20^ could be used to increase the number of addressable emitters while simplifying the fiber-to-chip connection. Lastly, we observed considerable variability in device thickness when thinning chips using the selected mechanical polishing post-processing method. This variability stems from the sensitivity of the final thickness to sample preparation and positioning, as well as the lack of feedback control for achieving a precise final device thickness. To reduce this variability in device thickness, more precise methods for device thinning or etching (e.g., deep reactive ion etching) should be explored.

In conclusion, we have demonstrated a foundry-fabricated, nanophotonic neural probe capable of dual-color photostimulation and electrophysiological recording. Through a combination of spatial and wavelength multiplexing, notably using on-shank robust directional-coupler wavelength demultiplexers and low-crosstalk waveguide arrays with dissimilar widths, a record 52 grating couplers (26 blue and 26 red) were integrated onto a single narrow (70  μm wide and 37  μm thick) neural probe shank, which also included 26 recording electrodes. Paths toward further increasing emitter density and improving optical transmission have been detailed. Our *in vivo* experiments in mice expressing red and blue light–sensitive opsins have demonstrated the functionalities of the neural probe while also highlighting the practicality of multi-probe experiments using photonic neural probes for photostimulation and CMOS-based probes for electrophysiological recording, each independently designed for either high photonic emitter or electrode density. Overall, this work provides a foundation for further scaling and maturation of dual-color photonic neural probes—toward higher emitter densities, larger optical output powers, and volume fabrication.

## 4 Methods

### 4.1 Neural Probe Fabrication

Neural probes were fabricated on 200-mm diameter Si wafers at AMF using a two-layer SiN visible-light integrated photonics platform with three Al metal layers.^27^ Fabrication began with the deposition of the silicon dioxide (${\mathrm{SiO}}_{2}$) waveguide bottom cladding. Next, 120-nm (SiN1) and 75-nm (SiN2) thick SiN waveguides and their 100-nm nominally thick interlayer ${\mathrm{SiO}}_{2}$ layer were defined by plasma-enhanced chemical vapor deposition, 193-nm-deep ultraviolet photolithography, and reactive ion etching; chemical mechanical polishing was used for planarization. Next, the ${\mathrm{SiO}}_{2}$ top cladding of the waveguides and three Al metal layers (M1, M2, and M3) with interconnecting vias were formed. Recording electrodes were coated with TiN. Deep trenches were etched to define the probe shapes and edge coupler facets. Finally, backgrinding was performed to thin the wafers to ∼100  μm and separate the chips.

### 4.2 Neural Probe Post-Processing and Laser-Induced Electrode Impedance Reduction

Neural probes were mechanically polished to reduce the probe shank thickness from ∼100 to 40  μm (NOVA Optical Polishing System, Krell Technologies Inc., Neptune City, New Jersey, United States). Samples were encased in mounting wax to avoid device damage during polishing. Samples were lowered at a rate of 5  μm every 30 s against a rotating 9  μm grit-size polishing film until a final thickness was reached. The thickness of neural probe samples was measured optically by imaging sample side profiles under a microscope with respect to a 1-mm measurement scale. All measurements were compared using Fiji.^46^ Following polishing, samples underwent at least two 16-h acetone bath treatments to reduce surface contamination from the mounting wax.

Laser-induced electrode impedance reduction was achieved using previously described methods.^18^ Briefly, a two-photon microscope (Bruker Corporation, Billerica, Massachusetts, United States) equipped with a femtosecond laser (Monaco, Coherent Corporation, Saxonburg, Pennsylvania, United States) was used to roughen electrode surfaces via selective laser scanning. The femtosecond laser repetition rate was set to 10 kHz, the wavelength was set to 1035 nm, and the average incident power was set to 60 to 80  μW. A laser scanning sequence was applied once per electrode, and repeated until the electrode surface appeared visibly darkened when viewed under an optical microscope [see Fig. 3(c), right].

### 4.3 Neural Probe Packaging

Flexible printed circuit boards (PCBs) were designed and fabricated (Würth Elektronik, Niedernhall, Germany) for connecting the neural probes to an external data acquisition system. Custom aluminum metal probe holders were designed and fabricated (Proto Labs Germany GmbH, Putzbrunn, Germany) for mounting the neural probes and flexible PCBs. Custom aluminum metal stereotactic adapters were designed and fabricated in-house by the Allen Institute for implanting the neural probes. Neural probe samples were mounted on the aluminum probe holder assemblies using metal epoxy (Loctite Ablestik 84-1LMIT1, Henkel AG & Co. KGaA, Düsseldorf, Germany), which was cured at 130°C for 4 h. Neural probe electrodes were then connected to the PCB via Al wire wirebonding, which were encapsulated in dielectric epoxy (Katiobond GE680, Delo, Germany). After electrical packaging, neural probe samples were connected to a custom 16-core multicore fiber (Corning Inc., Corning, New York, United States) using previously described methods.^18^ Briefly, multicore fibers were aligned to the neural probe edge couplers (each core coupled to an on-chip edge coupler) using a six-axis alignment stage and bonded using UV-curable optical epoxies (OP-67-LS and OP-4-20632, Dymax Corp., Torrington, Connecticut, United States). After curing, the optical epoxy was coated in an optically opaque epoxy (EPO-TEK 320, Epoxy Technology, Billerica, Massachusetts, United States) to reduce stray light emission.

### 4.4 Electrode Impedance Measurements

Electrode impedance values for neural probe chips were measured using an impedance analyzer (Keysight E4990A, Keysight Technologies, Santa Rosa, California, United States). Electrodes were lowered into a bath of 1× Dulbecco’s phosphate-buffered saline (DPBS) (Merck KGaA, Darmstadt, Germany), and electrode bond pads were connected to the impedance analyzer using a tungsten needle. The electrode impedance was measured using a 10-mV waveform relative to an Ag/AgCl electrode. After packaging, electrode impedances were measured using the impedance measurement function of an Intan RHD2132 headstage (Intan Technologies, Los Angeles, California, United States). Packaged neural probes were immersed in 1× DPBS, and electrode impedances were measured relative to an Ag/AgCl electrode.

### 4.5 Wavelength Demultiplexer Measurements

The optical transmission of wavelength demultiplexer test devices (on separate test chips) was measured using a supercontinuum laser (SuperK Extreme, NKT Photonics, Birkerød, Denmark) and an optical power meter (Newport, Irvine, California, United States). The supercontinuum laser output was polarized using a polarization filter, and the output polarization was maintained using a polarization-maintaining (PM) fiber. The output wavelength of the supercontinuum laser was set using a tunable optical bandpass filter (LLTF Contrast, NKT Photonics). A separate length-matched waveguide test structure was measured to subtract the loss of the edge couplers and routing waveguide from the demultiplexer test structures.

### 4.6 Beam Profile and Transmission Measurements

Neural probe optical beam profiles were measured in fluorescent solution to characterize the in-plane and side profile of the beam. The top profiles were measured using a vertically positioned fluorescence microscope (Objective: 10× Plan Apo, Mitutoyo Corporation, Kawasaki, Japan). Side beam profiles were measured using an additional horizontally positioned fluorescence microscope (Objective: 5× Plan Apo, Mitutoyo). Packaged neural probe samples were connected to a custom laser scanning system with a depolarized supercontinuum laser input (SuperK Fianium, NKT Photonics). The emission wavelength of the supercontinuum laser was selected using a tunable optical bandpass filter (LLTF Contrast, NKT Photonics). The laser scanning system used for the optical characterization of the probes was different from the dual-color scanning system used for *in vivo* experiments in this work (Sec. 4.7); having a single-mode fiber input and no free-space depolarizer. This simpler scanning system is detailed in our previous work.^47^ To characterize the blue grating coupler emitter, the optical input was set to 473 nm, and the probe was immersed in 100-μM fluorescein solution. Optical emission filters (ET525/50M, Chroma Technology Corp., Bellows Falls, Vermont, United States; BrightLine Basic Fluorescence Filter 525/39, IDEX Corporation, Northbrook, Illinois, United States) were inserted in the vertically positioned and horizontally positioned fluorescence microscopes, respectively, and the neural probe beam profiles were imaged (via imaging the fluorescence excited by the neural probe output). To characterize the red grating coupler emitter, the optical input was set to 638 nm and the probe was immersed in 2  mg/L Alexa Fluor 647 solution (Thermo Fisher Scientific, Waltham, Massachusetts, United States). Optical emission filters (FELH0650—650 nm long-pass filter, Thorlabs, Inc., Newton, New Jersey, United States) were inserted in the vertically positioned and horizontally positioned fluorescence microscopes for fluorescence imaging of the beam profiles. Probe transmission for three packaged nanophotonic probes was measured using the supercontinuum laser source and an optical power meter (Newport, Irvine, California, United States). An additional packaged nanophotonic probe, which was used in *in vivo* experiments, used the custom dual-color laser scanning system during transmission measurements.

### 4.7 Custom Dual-Color Laser Scanning System

A custom free-space laser scanning system [Figs. S2(a) and S2(b) in the Supplementary Material], similar to that described in Ref. 18, was designed for delivering high-intensity blue and red light to a multicore fiber coupled to the neural probe. A 300-mW 473-nm laser diode (Cobolt 06-MLD, Hübner Group, Kassel, Germany) and 180-mW 638-nm laser diode (Cobolt 06-MLD, Hübner Group) were directed through separately controlled variable neutral density filters (NDC-25C-2-A, Thorlabs, Inc., Newton, New Jersey, United States) and achromatic half-wave plates (AHWP10M-600, Thorlabs, Inc.) in rotation mounts. The beams were then combined using a dichroic mirror, and the combined beam was sent through a free-space Mach–Zehnder interferometer depolarizer with an optical path length difference of ∼220  mm. The depolarizer minimized the effects of polarization variability due to temperature and stress fluctuations in the multicore fiber, which additionally varies among cores (each core has a separate Jones matrix). As the neural probes function for both TE- and TM-polarized light (albeit with higher performance for the TE-polarization), the probes also function with depolarized light, which is effectively equal parts TE- and TM-polarized on-chip. After depolarization, the beam was reflected off a right-angle prism mirror and directed onto a MEMS mirror (Mirrorcle Technologies Inc., Richmond, California, United States). The MEMS mirror scanned the beam into a custom lens system, transferring the collimated beam to a focused spot for addressing the cores of the multicore fiber. The multicore fiber was positioned using a three-axis alignment stage (MBT616D/M, Thorlabs, Inc.). The MEMS mirror was controlled by a custom MATLAB GUI (MathWorks, Natick, Massachusetts, United States) to direct the beam at one of the 16 cores of the multicore fiber (i.e., the position of the MEMS mirror selected the fiber core and corresponding on-shank grating coupler pair to emit light). Pulse trains were defined via modulation of the red and blue lasers. The custom scanning optical system was originally designed for single-color operation, so in the current version, there is a chromatic focus shift between the red and blue laser wavelengths. Thus, for experiments, the axial position of the multicore fiber was positioned at either the blue-beam focus, red-beam focus, or an intermediate balanced defocus position between the blue- and red-beam foci depending on whether greater blue or red photostimulation intensities were required. Full chromatic correction can be achieved in the future by adding a diffractive optical element to the existing custom scanning optics design to maximize efficiency for red and blue light simultaneously.

### 4.8 Animal Experiments

All animal experiments were conducted under protocols approved by the Institutional Animal Care and Use Committee at the Allen Institute in Seattle, Washington. A VGAT-ChR2 mouse (male) (JAX #014548) and an Adora2a-Cre mouse (male) (MMRRC #36158) injected with AAV-flex-ChrimsonR (Addgene #62723)^9^ were used for assessing the functionalities of a packaged neural probe *in vivo*. Under deep isoflurane anesthesia, a titanium headframe was attached to the skull with Metabond (Parkell, Inc., Brentwood, New York, United States). Craniotomy and durotomy were performed over most of the left dorsal skull, which was replaced with a custom three-dimensional-printed cranial window.^48^ For the Adora2a-Cre mouse, 300 nL of virus (AAV-flex-ChrimsonR) was injected into the dorsal striatum at a coordinate of 0.7 mm posterior, 2.45 mm lateral, and 2.6 mm ventral to the bregma skull suture. Following at least 2 weeks of recovery and at least 24 h prior to recording, a protective layer of durable silicone (SORTA-Clear, Smooth-On, Inc., Macungie, Pennsylvania, United States) was replaced with a soft silicone elastomer (“Dura-Gel”, DOWSIL 3-4680) that would allow probes to penetrate into the brain.

Mice were trained to sit quietly while head-fixed in a 30-cm diameter plastic tube over the course of three habituation sessions. On the day of the recording, a nanophotonic probe and Neuropixels 2.0 single-shank probe were mounted on separate modules of a custom insertion system. Modules were placed at 4° from vertical along the anterior-posterior axis and either 0 to 5° (nanophotonic probe) or 25 to 32° (Neuropixels probe) along the medial-lateral axis. Each module included a three-axis manipulator (M3-LS-3.4-15, New Scale Technologies, Victor, New York, United States) that allowed each probe to be moved independently with sub-micron precision. Probes were visualized using a pair of long-working-distance microscopes (InfiniProbe, Infinity Photo-Optical, Centennial, Colorado, United States). Once the mouse was head-fixed, probes were aligned with holes in the cranial window so they would cross within 300  μm of one another in the target structure (retrosplenial cortex in the VGAT-ChR2 mouse and dorsal striatum in the Adora2a-Cre mouse). Probes were inserted into the brain at a rate of 200  μm/min. Electrophysiological data from the Neuropixels probe were recorded using a Neuropixels base station in a National Instruments PXI chassis. Electrophysiological data from the nanophotonic neural probes were digitized using a 32-channel recording headstage (RHD2132, Intan Technologies) connected to an Open Ephys acquisition board (Open Ephys Production Site, Lisbon, Portugal). All data were visualized and recorded using the Open Ephys GUI.^49^

### 4.9 Electrophysiological Data Analysis

Electrophysiological data were losslessly compressed using the WavPack algorithm^50^ and uploaded to an Amazon S3 storage bucket. Neuropixels data were processed using the SpikeInterface package^51^ running in the Allen Institute for Neural Dynamics Code Ocean computing environment. Data were high-pass filtered at 300 Hz and phase-shifted to align samples multiplexed into the same analog-to-digital converter, and a common median reference was applied across all channels. Data were sorted using Kilosort 2.5,^25^ followed by the calculation of standard quality metrics.^39^ All units included in the analysis had ≥200 spikes, inter-spike interval violation ratios <0.5, and median spike amplitudes >50  μV.

Electrophysiological data from the nanophotonic probe were analyzed using a custom electrophysiology processing pipeline in SpikeInterface^51^ at the Max Planck Institute of Microstructure Physics. Data were preprocessed by applying a band pass filter (300 to 6000 Hz) and a common median reference. To remove photostimulation artifacts, a 10-ms blanking period, centered on each stimulation pulse onset and offset, was applied. Spike sorting was completed using SpyKING CIRCUS2, an internal spike sorter available in SpikeInterface based on the SpyKING CIRCUS spike sorter.^52^ After sorting, spike and quality metrics were calculated for each unit. Units were manually curated in Phy.^53^ Units with less than 200 spikes and units with noise-like waveforms were excluded from the analysis. All units with similarity scores ≥0.9 were compared and considered for manual merging. All units included in the analysis had inter-spike interval violation ratios <0.5 and median spike amplitudes >50  μV.

### 4.10 Statistical Analysis

Average binned firing rate data from the *in vivo* experiments prior to and during blue and red photostimulation were compared with Mann–Whitney U rank tests using the SciPy Python package. Significance is indicated for p<0.05. All reported values are mean ± SD unless otherwise stated.

## Supplementary Material

10.1117/1.NPh.12.2.025002.s01

## Data Availability

Electrophysiological data reported in this study and code for analyzing data are available from David A. Roszko upon reasonable request.
