# Supplementary material for: Foundry-fabricated dual-color nanophotonic neural probes for photostimulation and electrophysiological recording
Source: Neurophotonics. 2025 Mar 28;12(2):025002. doi: 10.1117/1.NPh.12.2.025002 (PMC11952718; doi:10.1117/1.NPh.12.2.025002)
Supplement: Supplementary file 1 [file NPh_012_025002_SD001.pdf]

Supplementary Material

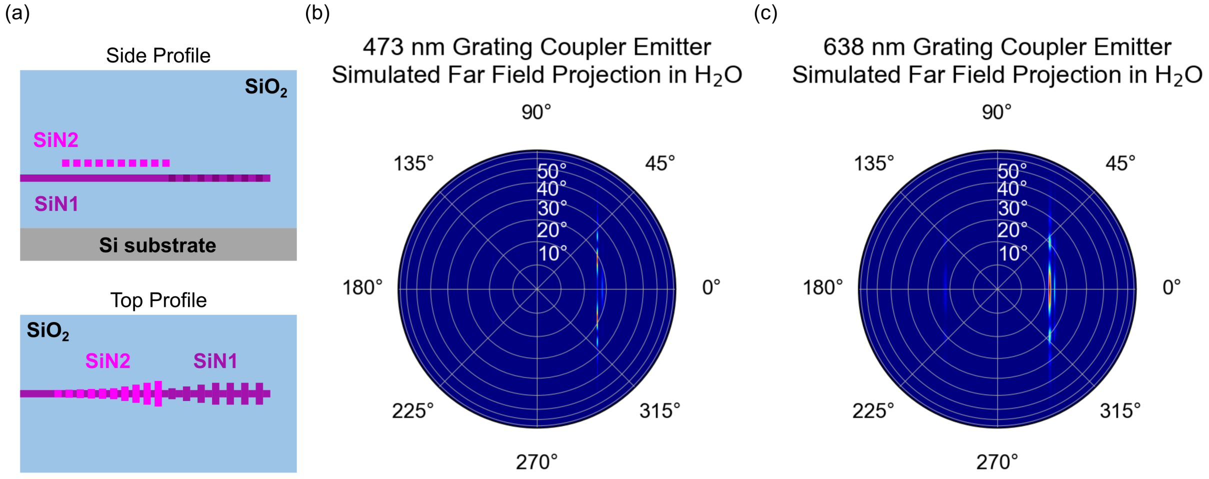

Fig S1 (a) Conceptual diagram of bi-layer grating coupler emitters. The design features a fully-etched grating coupler in SiN2, followed by a corrugated grating in SiN1. The 473 nm emitter (length: 101 μm) consists of a SiN2 grating (fill factor: 50%, period: 0.47 μm) with width apodization from 0.254 μm to 2.038 μm, followed by a SiN1 corrugated grating (fill factor: 50%, period: 0.47 μm) with central waveguide width of 0.22 μm and grating width apodization from 1.431 μm to 1.5 μm. The 638 nm emitter (length: 101 μm) consists of a SiN2 grating (fill factor: 50%, period: 0.63 μm) with width apodization from 0.493 μm to 1.381 μm, followed by a SiN1 corrugated grating (fill factor: 50%, period: 0.63 μm) with central waveguide width of 0.22 μm and grating width apodization from 0.627 μm to 1.26 μm. (b) Simulated far field projection of the 473 nm grating coupler emitter in water (refractive index, n = 1.3361). (c) Simulated far field projection of the 638 nm grating coupler emitter in water (refractive index, n = 1.3315). Simulations in (b,c) used Lumerical 3D FDTD with combined TE- and TM-polarized input light (modeling depolarized input light). The simulations are based on the designed waveguide and interlayer oxide thicknesses.

Table S1 Summary of average transmission data for the nanophotonic neural probe. Demultiplexer transmission data is calculated as the average transmission for TE- and TM-polarized light from test chip measurements (n=5). Nanophotonic probe transmission is calculated as the average transmission for TE- and TM-polarized light from test chip measurements (n=64 emitters from 3 chips). This value includes contributions from the demultiplexer transmission. Excess loss due to fiber packaging and fiber misalignment is estimated based on the transmission of packaged probe samples for depolarized light (n=78 emitters from 4 packaged probes) minus the nanophotonic probe transmission, where the laser scanning system is aligned to deliver maximum optical power at the given wavelength (see Methods 4.7). The laser scanning system coupling efficiency is based on MCF measurements when the system is aligned to deliver maximum optical power at the given wavelength (n=16 cores). The total system transmission is estimated as the sum of the laser scanning system coupling efficiency and the average transmission of packaged probe samples for depolarized light (n=78 emitters from 4 packaged probes). Data are reported as mean $\pm$ SD.

Laser	Total System Transmission [dB]	Laser Scanning System Coupling Efficiency [dB]	Estimated Excess Loss due to Fiber Packaging [dB]	Nanophotonic Probe Transmission [dB]	Demultiplexer Transmission [dB]
Blue (473 nm)	-31.5 $\pm$ 5.2	-4.4 $\pm$ 2.2	-13.2 $\pm$ 4.5	-13.9 $\pm$ 1.2	-0.6 $\pm$ 0.6
Red (638 nm)	-33.1 $\pm$ 4.5	-3.6 $\pm$ 1.2	-7.3 $\pm$ 4.2	-22.2 $\pm$ 0.9	-3.5 $\pm$ 1.7

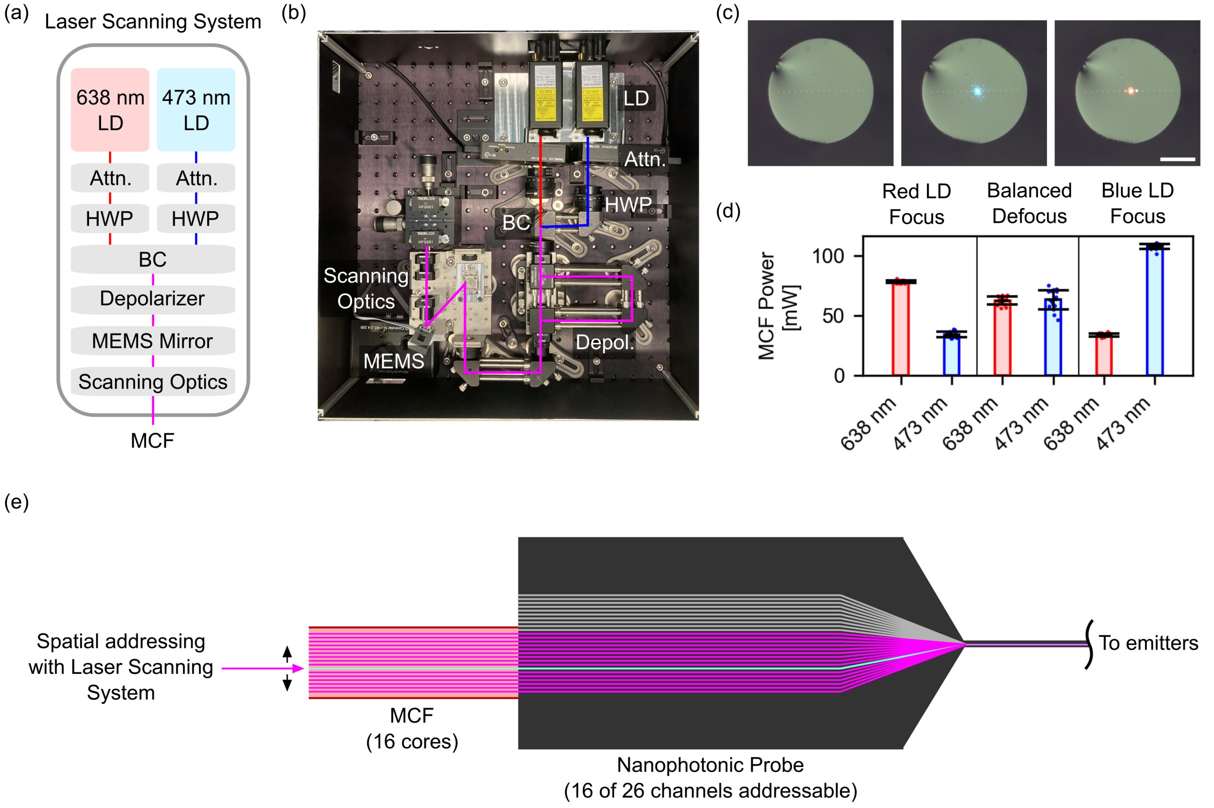

Fig S2 (a) Block diagram of custom dual-color laser scanning system. LD - laser diode, Attn. - variable attenuator, HWP - half-wave plate, BC - beam combiner, MEMS - microelectromechanical system, MCF - 16-core multicore fiber. (b) Labeled photograph of laser scanning system. (c) Micrographs of a 16-core multicore fiber facet without (left) and with blue/red (center/right) laser light coupled to a single core. Scale bar: 100 μm. (d) Average available output power from MCF connected to the laser scanning system as a function of MCF axial position. The MCF can be aligned to either the red LD or blue LD focal points for maximum power at a single wavelength, or can be aligned to a balanced defocused position for approximately equal red and blue optical power. (e) Schematic image of spatial addressing via 16-core multicore fiber. The multicore fiber used in this work permits 16 of the available 26 channels on the nanophotonic probe to be spatially addressed.

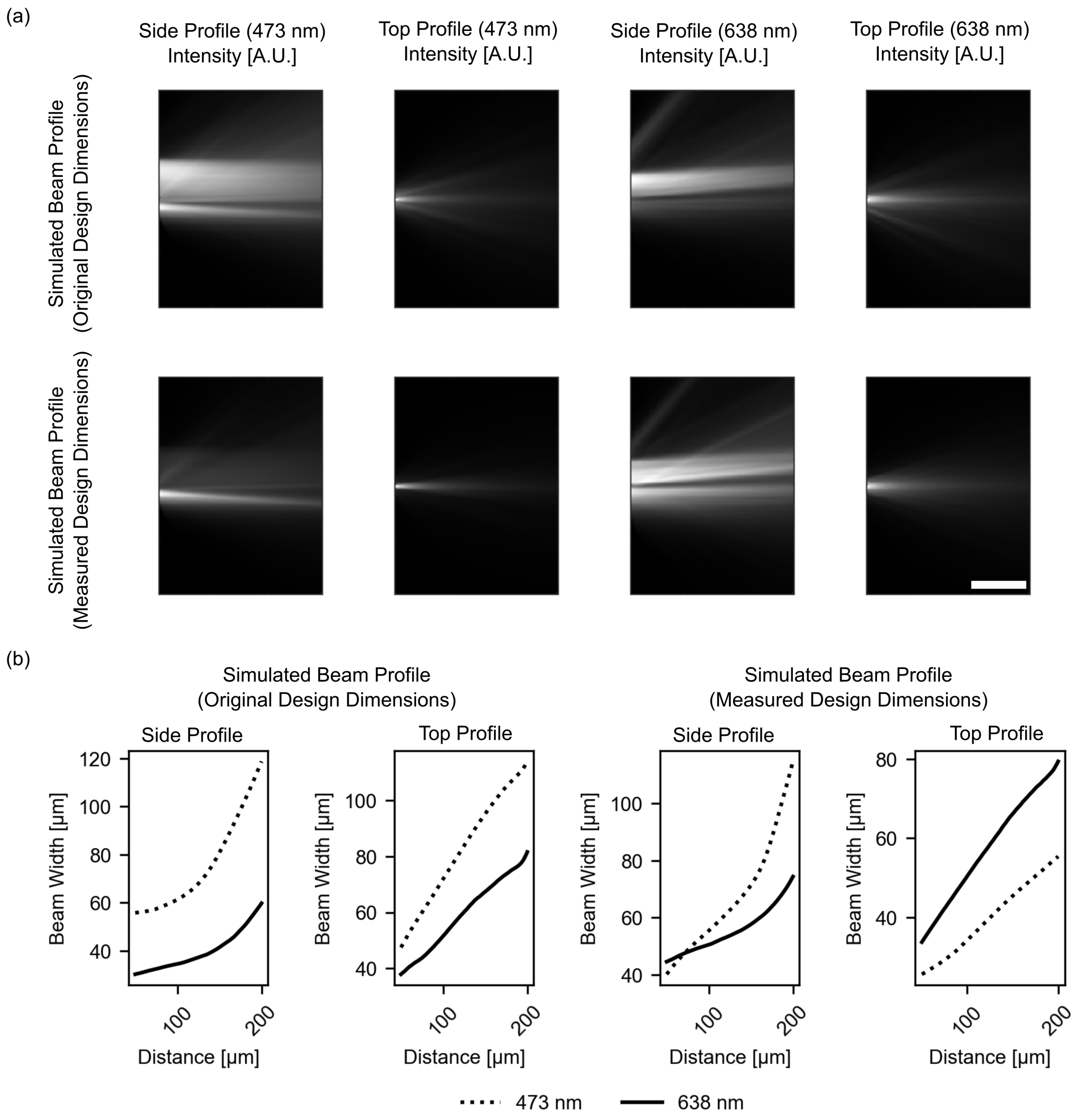

Fig S3 (a) Simulated beam profiles for red (638 nm) and blue (473 nm) grating coupler emitters. Beam profiles were simulated using the beam propagation method in scattering media.[31] Cross sectional images were constructed using methods outlined in Ref. 32. Top row: simulations based on original design parameters (see Methods). Bottom row: simulations based on updated layer thicknesses derived from cross-sectional transmission electron microscopy measurements. Scale bar: 50 μm. (b) Side profile and top profile FWHM beam widths of simulated beams 50 μm - 200 μm away from the emitter.

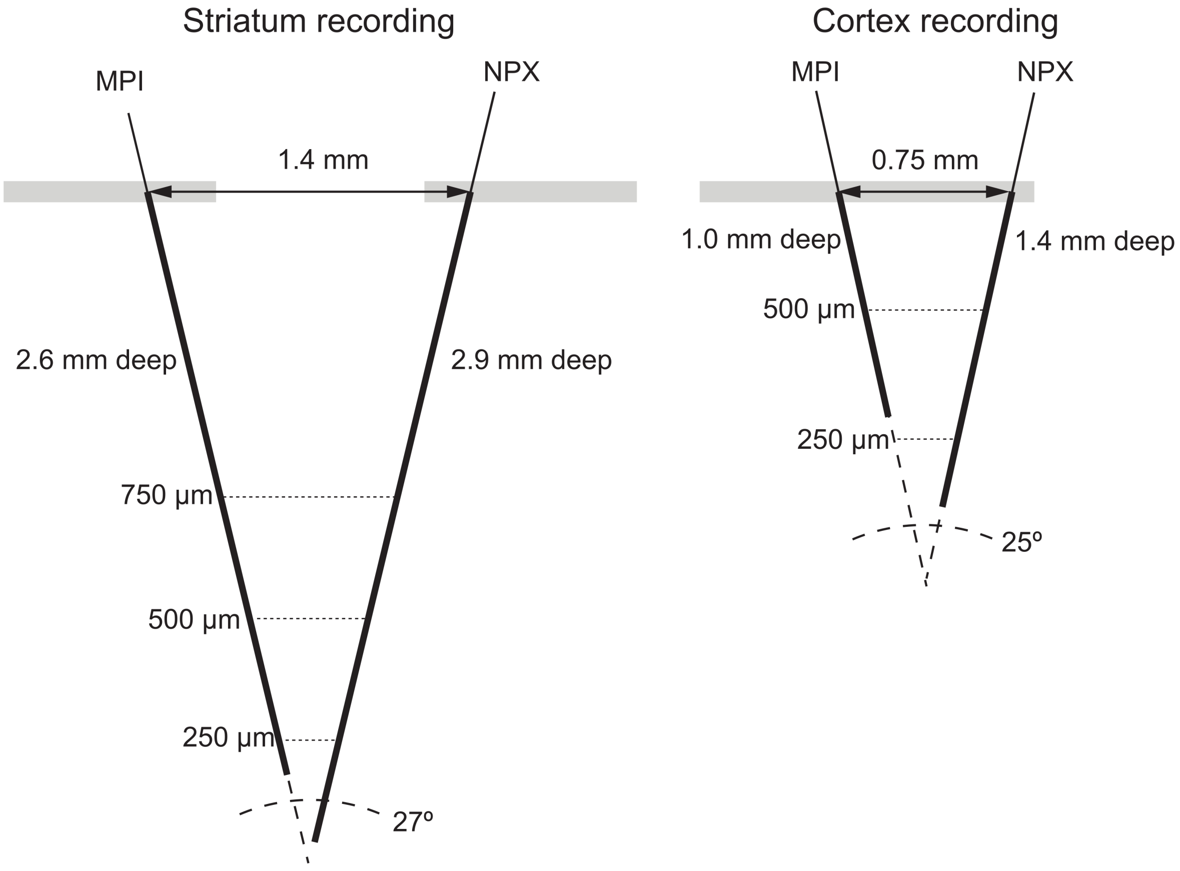

Fig S4 Relative positions of the nanophotonic probe (MPI) and Neuropixels probe (NPX) during *in vivo* experiments. Left: Striatum recording in red-light sensitive mouse (Adora2a-Cre mouse injected with AAV-flex-ChrimsonR). Right: Cortex recording in a blue-light sensitive VGAT-ChR2 mouse.

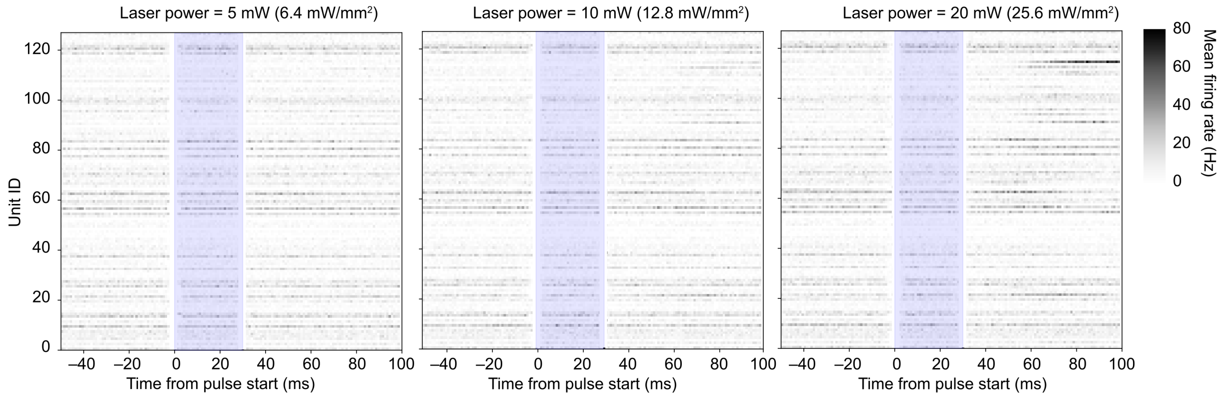

Fig S5 *In vivo* Neuropixels 2.0 recordings in cortex of a wild-type head-fixed mouse during photostimulation with a collimated 473 nm laser beam (approximate beam diameter: 1 mm). Laser power was adjusted from 5 mW to 20 mW. Each plot shows the average response over 600 pulses (60 trials, 10 pulses per trial). No network-wide decreases in firing rate were observed in response to photostimulation.

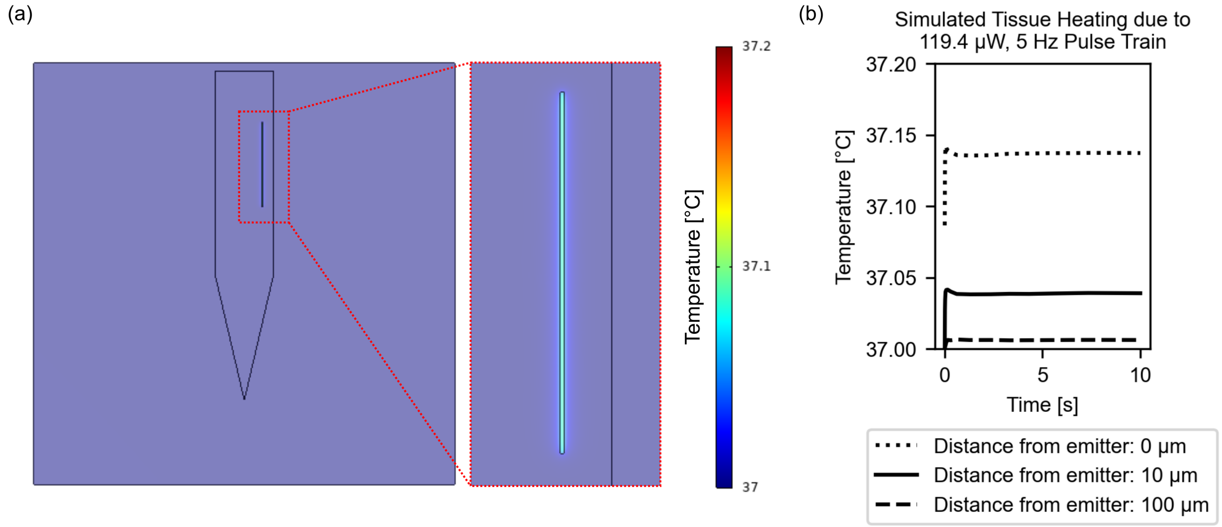

Fig S6 (a) COMSOL Multiphysics simulation of tissue heating due to photostimulation. Simulation parameters were derived from Ref. 18. The simulation presents a conservative estimate for tissue heating, where all optical power is directly absorbed at the emitter. The emitter power was set to 17.91 μW, equivalent to the average emitter power of the highest intensity pulse train used in this study (peak power: 119.4 μW, pulse width: 30 ms, pulse frequency: 5 Hz, effective duty cycle: 15%). (b) Time series data showing tissue temperature over time at various distances normal to the active emitter.

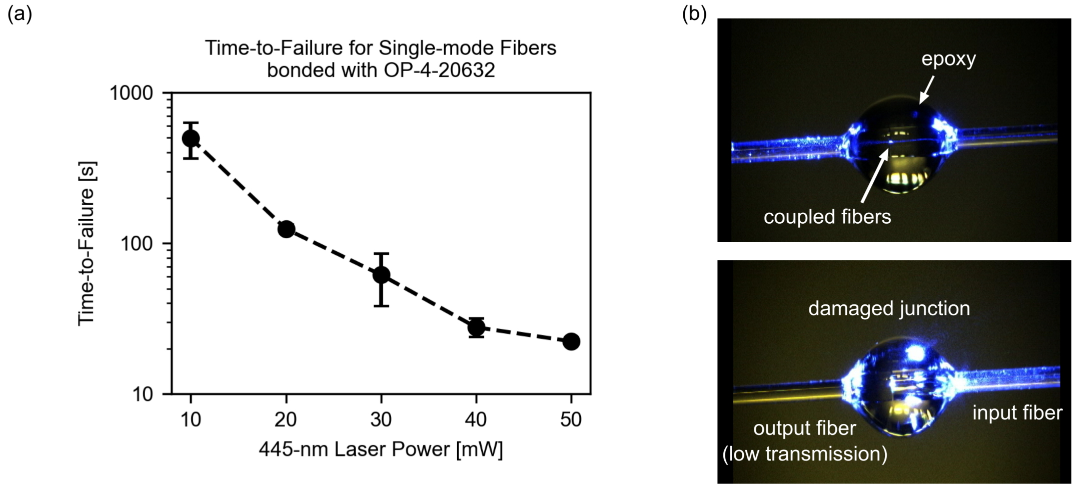

Fig S7 (a) Time-to-failure data as a function of input 445 nm laser power for single-mode fibers bonded with OP-4-20632 epoxy (n=3). (b) Photographs of bonded single-mode fibers before (top) and after (bottom) failure due to prolonged exposure to high-power laser light.
